# Loss of N‐WASP drives early progression in an Apc model of intestinal tumourigenesis

**DOI:** 10.1002/path.5086

**Published:** 2018-05-28

**Authors:** Hayley T Morris, Loic Fort, Heather J Spence, Rachana Patel, David F Vincent, James H Park, Scott B Snapper, Francis A Carey, Owen J Sansom, Laura M Machesky

**Affiliations:** ^1^ Cancer Research UK Beatson Institute Bearsden, Glasgow UK; ^2^ Academic Unit of Surgery, School of Medicine, Dentistry and Nursing University of Glasgow, Glasgow Royal Infirmary Glasgow UK; ^3^ Harvard Medical School and Boston Children's Hospital Division of Gastroenterology, Hepatology and Nutrition Boston Massachusetts USA; ^4^ Department of Pathology Ninewells Hospital Dundee UK; ^5^ Institute of Cancer Sciences University of Glasgow Bearsden, Glasgow UK

**Keywords:** N‐WASP, WASL, intestine, colon, adenoma, cancer

## Abstract

N‐WASP (WASL) is a widely expressed cytoskeletal signalling and scaffold protein also implicated in regulation of Wnt signalling and homeostatic maintenance of skin epithelial architecture. N‐WASP mediates invasion of cancer cells in vitro and its depletion reduces invasion and metastatic dissemination of breast cancer. Given this role in cancer invasion and universal expression in the gastrointestinal tract, we explored a role for N‐WASP in the initiation and progression of colorectal cancer. While deletion of N‐wasp is not detectably harmful in the murine intestinal tract, numbers of Paneth cells increased, indicating potential changes in the stem cell niche, and migration up the crypt–villus axis was enhanced. Loss of N‐wasp promoted adenoma formation in an adenomatous polyposis coli (Apc) deletion model of intestinal tumourigenesis. Thus, we establish a tumour suppressive role of N‐WASP in early intestinal carcinogenesis despite its later pro‐invasive role in other cancers. Our study highlights that while the actin cytoskeletal machinery promotes invasion of cancer cells, it also maintains normal epithelial tissue function and thus may have tumour suppressive roles in pre‐neoplastic tissues. © 2018 The Authors. *The Journal of Pathology* published by John Wiley & Sons Ltd on behalf of Pathological Society of Great Britain and Ireland.

## Introduction

Colorectal carcinoma (CRC) is the second most common cancer in Europe, with the second highest mortality rate 1. In spite of advances in diagnosis and management resulting in overall improved prognosis for patients with CRC, survival rates remain poor for those with advanced disease at presentation 2. Improved understanding of the pathogenesis and progression of CRC is therefore desirable, in order to mediate early diagnosis and prevention of metastasis.

N‐WASP [neural Wiskott–Aldrich syndrome protein, also termed Wiskott–Aldrich syndrome‐like (WASL)] is a key organiser of invadopodia, actin‐based structures involved in matrix remodelling 3, 4, 5, 6. Depletion of N‐WASP in breast cancer cells decreased invasion and matrix remodelling 6 and led to decreased motility and metastasis 7. Thus, N‐WASP is potentially a key target for invasion and metastasis of epithelial cancers. In addition to its role in cancer, N‐WASP has a scaffolding role in cell–cell adhesion and signalling in epithelial tissues. N‐WASP is a positive regulator of Wnt pathway signalling in skin 8 and hair follicle cycling through TGF‐beta signalling 9. N‐WASP promotes the integrity of barriers, such as the blood–testis barrier 10, in kidney podocyte foot processes 11 and in vascular permeability in the lung 12. N‐WASP also mediates myoblast cell–cell fusion during myogenesis 13. Thus, N‐WASP is important for normal tissue architecture and integrity, so careful consideration must be given to whether disruption of N‐WASP in cancer would have a positive or a negative effect on progression.

The few studies of N‐WASP in human cancers have shown a correlation between increased expression of N‐WASP and poor prognosis and/or advanced stage in pancreatic adenocarcinoma 14, hepatocellular carcinoma 15, oesophageal squamous carcinoma 16, and invasive ductal carcinoma of the breast 6. Conversely, there was decreased expression in clear cell renal cell carcinoma, but a high level of N‐WASP correlated with poorer survival 17. In many human cancer studies, high N‐WASP expression was negatively correlated with survival 14, 15, 17. N‐WASP levels were higher in metastatic liver lesions than in primary colonic tumours 18. Furthermore, the chromosomal locus of *N‐WASP*, 7q, is amplified in 22% of colonic adenomas and 44% of carcinomas 19. More recently, a genome‐wide association study identified several new susceptibility loci for CRC, including 1p36.12, the gene locus of *CDC42*, an upstream regulator of N‐WASP 20. Therefore, N‐WASP is a promising target for investigation in CRC.

To explore the role of N‐WASP in intestinal homeostasis or CRC, we deleted *N‐wasp* in a mouse model involving loss of *Apc* (adenomatous polyposis coli), either homozygously in the short term (3–4 days) or heterozygously, to drive adenoma progression. We also deleted *N‐wasp* in a more rapid model with *Apc* loss in combination with *KRas* ^G12D^, which are common genetic alterations driving human CRC 21. Additionally, we explored the relationship between N‐WASP protein levels and a range of clinicopathological parameters in two cohorts of human CRC (early polyp cancers and more advanced cancers). We present new evidence for a role for N‐WASP in the regulation of intestinal epithelial differentiation and indicate that it may act as a tumour suppressor against the development of benign precursor lesions of CRC (adenomas).

## Materials and methods

### Ethics and guidelines

All mouse experiments were compliant with the Animals (Scientific Procedures) Act and UK Home Office Regulations, and conducted with reference to the National Centre for the Replacement, Refinement and Reduction of Animals in Research guidelines 22.

All work involving human samples was carried out in accordance with national and local guidelines and with reference to the REMARK guidelines 23. Ethical approval for the Tayside whole tissue blocks was obtained from the Tayside Tissue Bank (request number TR000312). For the Glasgow TMAs, ongoing ethical approval for the use of the TMAs is held by the GRI Academic Unit of Surgery (Research Ethics Committee reference numbers 09/S0703/59 and 12/WS/0152).

### Murine models

Mice were bred on a C57BL/6 background and maintained in a pathogen‐free unit; experimental cohorts were moved to a non‐barrier unit and acclimatised for at least 5 days before induction and monitoring. All mice were maintained on a standard diet of Special Diets and Services (SDS, product code 801730) and water *ad libitum*. Expression of mutations was controlled by a tamoxifen‐inducible, gut‐specific promotor, *Villin*‐Cre‐ER^T2^ 24, combined with the following alleles: *Apc* ^fl^ (Apc580s, MGI: Apctm1Tno) 25, *KRas* ^G12D^ (MGI: KRastm4Tyj) 26, and *N‐wasp* ^fl^ (MGI: Wasltm2Sbs) 27. Controls in the long‐term *N‐wasp* knockout model were a mix of genotypes, with details of all cohorts provided in the supplementary material, Tables S1–S3.

### Tamoxifen induction

Mice were induced when they reached 20 g weight (age range 6–18 weeks across all cohorts; see supplementary material, Tables S1–S3) with intraperitoneal injections of tamoxifen [1 g of tamoxifen powder (Sigma‐Aldrich, Dorset, UK; Cat. No. T5648) resuspended in 10 ml of 100% ethanol (VWR Chemicals, Lutterworth, Leics, UK; Cat. No. 20821.330) and 90 ml of corn oil (Sigma‐Aldrich; Cat. No. C8267) to give a 10 mg/ml solution]. Mice received two 80 mg/kg doses, 24 h apart, except mice carrying the *KRas* ^G12D^ mutation, which received one injection. The time of the first dose was taken as ‘day 0’.

### Mouse sampling

In the short‐term models, mice were sacrificed on either day 3 (mice carrying the *KRas* ^G12D^ mutation) or day 4 (mice with wild‐type *KRas*) following induction. Mice reaching the endpoint were sacrificed, and the small intestine and colon were removed, flushed with ice‐cold water, opened longitudinally, and pinned, with the luminal surface facing upwards, on a wax‐filled Petri dish. Tissue was fixed in formalin for at least 24 h. Tumour counts and measurements were all performed at the endpoint (see supplementary material, Table S3).

### Tissue processing

Following fixation, the tissue was embedded in paraffin and sections were cut and stained as indicated. Antibody details are provided in Table 1. For bromodeoxyuridine (BrdU) labelling, 2 or 24 h prior to sacrifice, mice were injected with 250 μl of BrdU (GE Healthcare, Little Chalfont, UK; Cat. No. RPN201) to label dividing cells and stained with anti‐BrdU antibody.

**Table 1:** Details of the antibodies used for immunohistochemistry

Antibody	Supplier	Cat. No.	Dilution	Retrieval
Ki67 (SP6) rabbit monoclonal	Thermo Scientific, Renfrew, UK	RM‐9106‐S	1:100	PT Module pH 6 sodium citrate (98 °C)
Synaptophysin (SY38) mouse monoclonal	AbCam, Cambridge, UK	Ab8049	1:75	PT Module pH 6 sodium citrate (98 °C)
p‐Erk1/2 (Thr 202/Thy 204) rabbit polyclonal	Cell Signaling, Leiden, The Netherlands	9101	1:400	PT Module pH 6 sodium citrate (98 °C)
Lysozyme rabbit polyclonal	Dako, Ely, UK	A099	1:1000	Proteinase K solution (10 min RT)
Caspase 3 (ASP‐175) rabbit monoclonal	Cell Signaling, Leiden, The Netherlands	9661	1:50	PT Module pH 6 sodium citrate (98 °C)
BrdU mouse monoclonal	BD Biosciences, Wokingham, UK	347580	1:200	PT Module pH 6 sodium citrate (98 °C)
N‐WASP rabbit polyclonal	Sigma‐Aldrich, Doset, UK	HPA 005750	1:350 (mouse) 1:200 (human)	PT Module pH 6 sodium citrate (98 °C)

RT = room temperature.

### Quantification

Images were taken using the Olympus BX51 brightfield microscope, to visualise at least 100 well‐oriented half‐crypts per anatomical site per mouse. Quantification was performed manually using the cell counter tool in ImageJ.

### Human samples

The clinical data linked to both of the Glasgow TMAs were collected and maintained by the GRI Academic Unit of Surgery. The early polyp cancer TMA and the advanced cancer TMA are curated by David Mansouri and James Park, respectively, both Clinical Lecturers in Surgery, under the supervision of Professor Donald McMillan, Professor of Surgical Science.

Whole tissue blocks of normal small intestine and colon, adenoma, and adenocarcinoma were obtained from the Tayside Tissue Bank. Human colorectal cancer TMAs were obtained from the Glasgow Royal Infirmary (GRI) Academic Unit of Surgery. The early polyp cancer TMA contains cores from 182 patients (three cores per tumour) who underwent resection of early T1/T2 polyp cancers as part of the NHS Bowel Screening Programme. The advanced cancer TMA contains cores from 272 patients (four cores per patient) who underwent surgery for stage I–III colorectal cancer between 1997 and 2005.

TMA slides were scanned digitally at ×20 magnification using the high‐resolution Hamamatsu NanoZoomer HT (Hamamatsu Photonics, Shizuoka, Japan) and uploaded onto the SlidePath Digital Image Hub 4.0 (Leica Biosystems, Buffalo Grove, IL, USA). N‐WASP IHC for both TMAs was scored manually using the weighted histoscore method 28. Images were viewed at ×20 magnification using the SlidePath Hub and scores recorded within the OpTMA window. Each core was assigned a score based on the percentage of cells demonstrating varying degrees of positive N‐WASP staining within the cytoplasm (0 = negative, 1 = weak, 2 = moderate, 3 = strong – see supplementary material, Figure S6A).

### Analysis of publicly available datasets

To analyse *N‐WASP* (*WASL*) mRNA expression in human CRC, the TCGA (Provisional) dataset of colorectal cancer was examined utilising Oncomine and cBioPortal. Using the filters Gene: WASL, Analysis Type: Cancer vs. Normal Analysis, and Cancer Type: Colorectal Cancer, expression values were extracted from Oncomine and analysed in Prism. From the cBioPortal homepage, the query was run by selecting cancer study ‘Colorectal Adenocarcinoma (TGCA, Provisional)’ and genomic profiles ‘Putative copy‐number alterations from GISTIC’ and ‘mRNA Expression *z*‐scores (RNA Seq V2 RSEM)’, then entering ‘WASL’ within the ‘Enter gene set’ box. Graphs were generated by navigating to the ‘plots’ tab and selecting to plot ‘Putative copy‐number alteration’ against ‘mRNA Expression *z*‐scores (RNA seq V2 RSEM)’. After analysing the resulting graph, the query was repeated using a *z*‐score threshold of −1.2 as the cut‐off to define cases of altered gene expression (WASL:EXP < −1.2) and the ‘survival curve’ tab selected to display the resulting Kaplan–Meier curves for overall and disease‐free survival.

### Statistics

All statistical analyses and graphs were created using Prism 5 for Max OS X (GraphPad Software, Inc, San Diego, CA, USA), except the human colorectal TMA data, which were analysed using IBM® SPSS® Version 22 (IBM Corporation, Armonk, NY, USA), and graphs based on these data and those exported from cBioPortal were generated using R version 3.3.2 (The R Foundation for Statistical Computing). Unless stated otherwise, comparisons between groups were performed using a Mann–Whitney test. All *P* values are unadjusted. In the tumour model, mice culled for a reason other than reaching the endpoint for the model were included in the survival analysis as censored data.

## Results

### N‐WASP is not required for short‐term homeostasis in the intestine or colon

**Figure 1:**
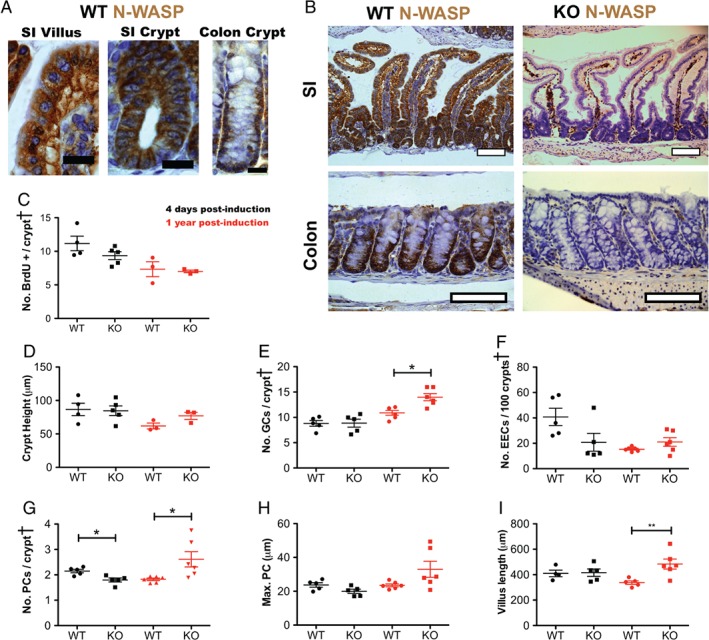
N‐WASP knockout in the intestine. (A) Representative images of N‐WASP IHC in normal mouse (WT) small intestine (SI) villi and crypts and colon. Scale bars = 20 μm. (B) Representative images of N‐WASP IHC in normal (WT) mouse intestine (upper left) and colon (lower left), and 3 days post‐tamoxifen induction in N‐wasp ^fl/fl^ (KO) intestine (upper right) and colon (lower right). Scale bars = 100 μm. Graphs C–I show time points at 4 days (black) and 1 year (red) post‐tamoxifen induction in wild‐type (WT) and N‐wasp ^fl/fl^ (KO) intestine as indicated. (C) Number of BrdU‐positive cells per half crypt/villus unit. (D) Intestinal crypt height (measured as distance of highest BrdU‐positive cell from crypt base). (E) Number of goblet cells (GCs) per half crypt/villus unit. (F) Number of enteroendocrine cells (EECs) per 100 half crypt/villus units. (G) Number of Paneth cells (PCs) per half crypt/villus unit. (H) Position of highest Paneth cell (measured as distance from crypt base). (I) Length of crypt/villus (measured from crypt base to villus tip). All graphs: n = 3–6 mice, counting 100 half crypts/villi per sample. Error bars = SEM. *p < 0.05 (Mann–Whitney). ^†^Crypt = half crypt/villus unit.

N‐WASP is expressed along the entire length of the epithelium of mouse intestine and colon and appears cytoplasmic and membrane‐associated (Figure 1A, B). Deletion of N‐WASP using *Villin*‐Cre‐ER^T2^ *N‐wasp* ^fl/fl^ mice did not alter intestinal or colonic tissue morphology despite a near‐complete absence of N‐WASP staining by day 3 (Figure 1B). Loss of N‐WASP did not affect proliferation at 4 days post‐tamoxifen induction (Figure 1C, D, black dots). The lowest BrdU‐positive cell was around 10 μm from the crypt base, regardless of *N‐wasp* status (*p =* 0.11) (supplementary material, Figure S1A), and the highest (crypt height) at just over 80 μm (*p =* 0.73) (Figure 1D). There was also no difference in the number or position of BrdU‐positive cells in the colon (supplementary material, Figure S1B, C).

In general, specialised cell types were unaffected by N‐WASP loss by day 4 after induction, as the number of goblet cells [GCs, Alcian blue and periodic acid‐Schiff (ABPAS)‐positive] (Figure 1E, black dots), enteroendocrine cells (EECs, synaptophysin‐positive) (Figure 1F, black dots), and Paneth cells (PCs, lysozyme‐positive) (Figure 1G, black dots) was unchanged. Localisation of Paneth cells within the crypt–villus axis (assessed by measuring the height of the highest lysozyme‐positive cell from the crypt base) by day 4 following induction (Figure 1H, black dots) was also unchanged. There was also no effect on apoptosis (cleaved caspase‐3‐positive) (supplementary material, Figure S1D, black dots) or villus length (Figure 1I). Thus, N‐WASP is expressed throughout the epithelium of the intestine and colon and its loss does not affect morphology, proliferation, apoptosis, or differentiation or distribution of specialised cell types in the short term.

At 1 year post‐induction, N‐WASP depletion is sustained, although focal expression was observed (supplementary material, Figure S1E). Proliferation (Figure 1C, D and supplementary material, Figure S1A–C, red dots) and apoptosis (supplementary material, Figure S1D, red dots) were unaffected at 1 year following N‐WASP loss. However, small but statistically significant increases in both Paneth and goblet cell numbers, but not enteroendocrine cell numbers or position of the highest Paneth cell, were observed (Figure 1E–H, red dots). There was also an increase in villus length (Figure 1I, red dots) from 338.0 ±13.73 to 483.5 ±39.30 (*p =* 0.0105). Thus, we conclude that loss of N‐WASP causes only minor perturbations of normal intestinal epithelial homeostasis and can be sustained up to 1 year.

### Loss of N‐WASP results in increased intestinal epithelial cell migration

**Figure 2:**
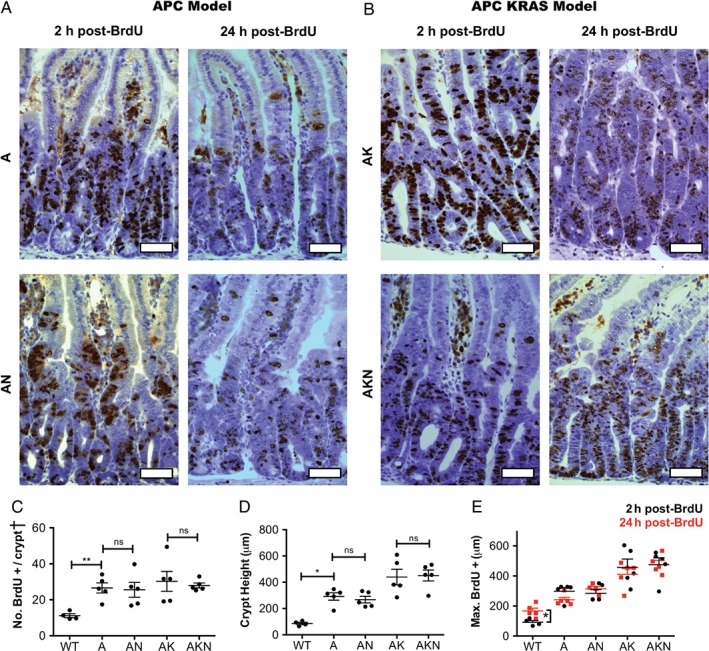
N‐WASP knockout does not affect intestinal epithelial proliferation in a rapid model. (A) IHC for BrdU at 2 and 24 h following intraperitoneal BrdU injection in Apc ^fl/fl^ (A) and Apc ^fl/fl^ N‐wasp ^fl/fl^ (AN) intestines. Scale bars = 50 μm. (B) IHC for BrdU at 2 and 24 h in Apc ^fl/fl^ KRas ^G12D/+^ (AK) and Apc ^fl/fl^ KRas ^G12D/+^ N‐wasp ^fl/fl^ (AKN) intestines. Scale bars = 50 μm. (C) Number of BrdU‐positive cells per half crypt/villus unit as indicated. (D) Intestinal crypt height (measured as distance of highest BrdU‐positive cell from crypt base). (E) Cell migration along the crypt–villus axis as assessed by change in position of the highest BrdU‐positive cell at 2 h (black) and 24 h (red) as indicated. All graphs: n = 4–5 mice, counting 100 half crypts/villi per sample. Error bars = SEM. *p < 0.05 (Mann–Whitney). ^†^Crypt = half crypt/villus unit.

Normally, epithelial cells of the intestine are in constant flux from the crypt area, where they arise up toward the top of the villus and eventually slough off 29, 30. This movement is referred to as migration along the crypt–villus axis and is driven by proliferation, but also involves differential expression of integrins and matrix ligands along the axis 29, 30. We tested the effect of *N‐wasp* deletion on this migration by treating mice with BrdU and measuring the position of the cells along this axis at 2 and 24 h post‐injection. Loss of *N‐wasp* accelerated the migration of BrdU‐labelled cells both in young and in older mice (supplementary material, Figure S1F, G). Deletion of *Apc* abrogates the migration of epithelial cells along the intestinal crypt–villus axis 31, and in the context of homozygous *Apc* deletion (Figure 2A) or *Apc* deletion combined with additional *KRas* ^G12D^ mutation (Figure 2B), additional loss of *N‐wasp* did not accelerate migration (Figure 2E). Loss of *Apc* also increases proliferation and crypt height 31, neither of which was affected by additional loss of *N‐wasp* (Figure 2C, D). Thus, the effect of *Apc* deletion was stronger than that of *N‐wasp* deletion. Loss of *N‐wasp* had no effect on proliferation in the colon (supplementary material, Figure S2A, B).

### Loss of N‐WASP alters differentiation and migration in a model of short‐term homozygous Apc deletion

**Figure 3:**
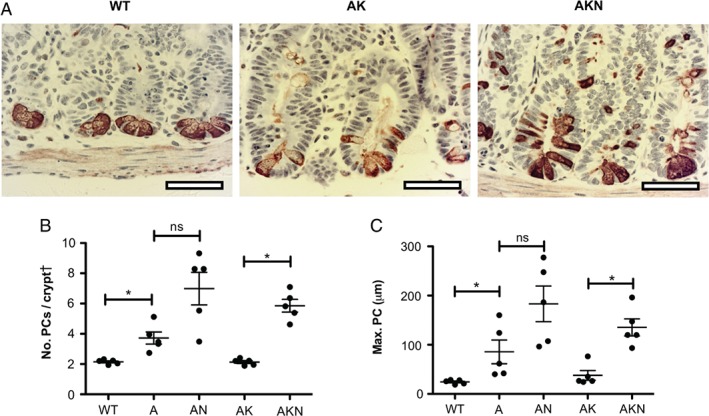
Effect of N‐WASP knockout on small intestinal Paneth cells in a rapid model at 4 days post‐induction. (A) Representative images of IHC for lysozyme to identify Paneth cells in wild‐type (WT), Apc ^fl/fl^ KRas ^G12D/+^ (AK), and Apc ^fl/fl^ KRas ^G12D/+^ N‐wasp ^fl/fl^ (AKN) mice. Scale bars = 50 μm. (B) Number of Paneth cells (PCs) per half crypt/villus unit as indicated. (C) Position of highest Paneth cell (measured as distance from crypt base) as indicated. For B and C: n = 4–5, counting 100 half crypts/villi per sample. Error bars = SEM. *p < 0.05. ns = not significant (Mann–Whitney). ^†^Crypt = half crypt/villus unit.

Homozygous loss of *Apc* in the intestine is lethal, but in the short term extensive hyperproliferation caused by total *Apc* loss can be studied up to 4 days past induction 31. Loss of *Apc* increases the number of Paneth cells and the height of their spread along the crypt–villus axis 31 (Figure 3A–C). Loss of *N‐wasp* increased the number and position of Paneth cells in the crypt–villus axis, on a background of combined loss of *Apc* with expression of *KRas* ^G12D^ (Figure 3A–C). Interestingly, this is similar to, but even more marked than, the alterations in Paneth and goblet cell numbers seen in *N‐wasp* ^fl/fl^ mice at 1 year post‐induction (Figure 1E, G, H). Loss of *Apc* slightly decreased the number of goblet cells, but this was restored by loss of *N‐wasp* (supplementary material, Figure S2C). This effect was negligible in the presence of *KRas* ^G12D^ (supplementary material, Figure S2C). There was no effect of *N‐wasp* deletion on the number of enteroendocrine cells (supplementary material, Figure S2D) or apoptotic cells (supplementary material, Figure S2E) in any of the genotypes. Thus, *N‐wasp* loss promoted increased Paneth cell numbers, which potentially could affect the Lgr5^+^ stem cell niche 32, 33.

### N‐Wasp knockout increased tumour burden and decreased survival in an Apc‐deficient model of tumourigenesis

**Figure 4:**
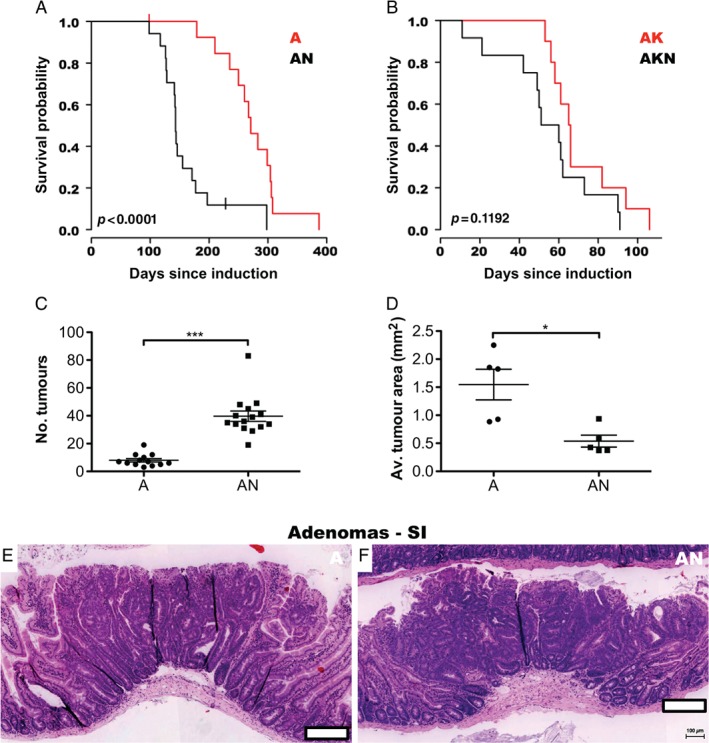
Loss of N‐WASP accelerates an Apc heterozygous loss model of intestinal tumourigenesis. (A) Kaplan–Meier survival curves (days since induction) for Apc ^fl/+^ (A, red line, n = 14) and Apc ^fl/+^ N‐wasp ^fl/fl^ (AN, black line, n = 17) mice. P value derived from log‐rank test. (B) Kaplan–Meier survival curves (days since induction) for Apc ^fl/+^ KRas ^G12D/+^ (AK, red line, n = 10) and Apc ^fl/+^ KRas ^G12D/+^ N‐wasp ^fl/fl^ (AKN, black line, n = 12) mice. P value derived from log‐rank test. (C) Total intestinal tumour counts for Apc ^fl/+^ (A, n = 13) and Apc ^fl/+^ N‐wasp ^fl/fl^ (AN, n = 15) mice. Error bars = SEM. ***p < 0.001 (Mann–Whitney). (D) Average intestinal tumour area in Apc ^fl/+^ (A) and Apc ^fl/+^ N‐wasp ^fl/fl^ (AN) mice. n = 5. Error bars = SEM. *p < 0.05 (Mann–Whitney). (E) Representative image of intestinal tumour from Apc ^fl/+^ mouse. Scale bar = 200 μm. (F) Representative image of intestinal tumour from Apc ^fl/+^ N‐wasp ^fl/fl^ mouse. Scale bar = 200 μm.

Heterozygous deletion of *Apc* drives the formation of adenomas (benign tumours) and microadenomas (very small tumours not visible to the naked eye – examples of histology are provided in the supplementary material, Figure S4A) in the small intestine of mice with a long latency; combining *Apc* loss with expression of mutant *KRas* ^G12D^ accelerates this progression by around three‐ to four‐fold 34, 35. Heterozygous *Apc* deletion induced tumours in mice with a median survival of around 271 days, but *N‐wasp* loss accelerated progression (median survival 143 days; Figure 4A, black line). Addition of *KRas* ^G12D/+^ accelerated progression further (median survival 65.5 days), which was not significantly shortened by loss of *N‐wasp* (55.5 days) (Figure 4B, black line). Loss of *N‐wasp* together with heterozygous *Apc* loss resulted in significantly more tumours in the small intestine by the endpoint (Figure 4C). However, in the colon, there was a small decrease in the number of tumours in the *Apc* ^fl/*+*^ *N‐wasp* ^fl/fl^ mice (supplementary material, Figure S3A). Although *Apc* ^fl/+^ *N‐wasp* ^fl/fl^ mice showed more intestinal tumours compared with *Apc* ^fl/+^ controls, the average tumour size was reduced (Figure 4D). There was no significant N‐WASP‐dependent difference in the size of colonic tumours (supplementary material, Figure S3B) or Ki67 (proliferation marker) expression (supplementary material, Figure S3C). The number and size of intestinal tumours in *Apc* ^fl/+^ *KRas* ^G12D/+^ mice were independent of *N‐wasp* status (supplementary material, Figure S3D, E); however, there was a small but significant decrease in the number of tumours in the colon (supplementary material, Figures S3F, G). There were no morphological differences between tumours in *Apc* ^fl/+^ and *Apc* ^fl/+^ *N‐wasp* ^fl/fl^ intestinal and colonic tumours (Figure 4E, F and supplementary material, Figure S4A, B). Thus, loss of *N‐wasp* accelerated the effects of *Apc* depletion by increasing the number of tumours. This suggests a possible tumour suppressor function for N‐WASP in the early stages of colorectal cancer.

### N‐WASP expression is correlated with disease‐free survival in human colorectal cancer

**Figure 5:**
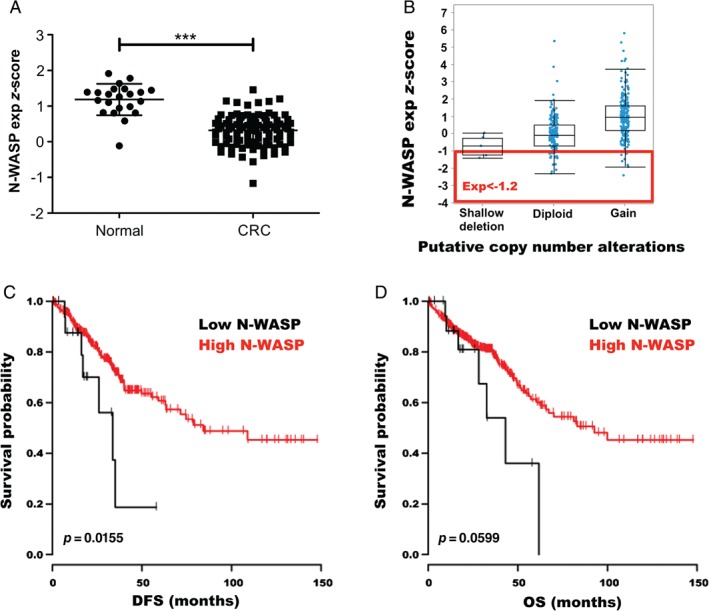
N‐WASP in human colorectal cancer – online data mining. (A) N‐WASP (WASL) mRNA expression z‐score levels in colorectal cancer (CRC) compared with control tissue (normal). Error bars = SEM. ***p < 0.001 (Mann–Whitney). (B) mRNA expression z‐scores of N‐WASP (WASL) in the TCGA (Provisional) dataset by copy number status. −1.2 was chosen as the cut‐off for low N‐WASP expression. (C, D) Survival curves for patients from the TCGA dataset stratified by N‐WASP (WASL) mRNA z‐score versus (C) disease‐free survival (DFS) and (D) overall survival (OS). Black lines represents patients with N‐WASP mRNA z‐score ≤ 1.2 (low expression) and red lines represents cases with z‐score ≥ 1.2. (normal/high expression). P value derived from log‐rank test.

Data obtained from Oncomine™ (Compendia Bioscience, Ann Arbor, MI, USA) showed significantly lower expression of *N‐WASP* mRNA in cancer versus normal tissue for 7/12 CRC datasets using a cut‐off of *p* ≤ 0.01, 1.5‐fold, and the top 10% of underexpressed genes. Specifically, in the TCGA (Provisional) cohort, there was a statistically significant decrease in the expression of *N‐WASP* mRNA in CRC versus normal colorectal tissue (*p* < 0.001, Figure 5A).

The TCGA Provisional (http://cancergenome.nih.gov/) dataset was also examined using cBioPortal (Figure 5B) and we generated Kaplan–Meier survival curves of patients with low (*z*‐score < −1.2) and normal/high (*z*‐score ≥ −1.2) *N‐WASP* mRNA expression. In this dataset, low *N‐WASP* expression was associated with a statistically significant decrease in disease‐free survival (*p =* 0.016) (Figure 5C) and a trend towards poorer overall survival (Figure 5D), although not statistically significant (*p =* 0.06).

### N‐WASP expression is correlated with differentiation and possible T stage in human early‐stage polyp cancers

Tissue from the Tayside Tissue Bank revealed that N‐WASP was largely cytoplasmic in human small intestine (supplementary material, Figure S5A) and colon (supplementary material, Figure S5B), and was expressed at moderate to high levels in the epithelium throughout the length of the crypt–villus axis. Likewise, N‐WASP showed variable intensity in human benign adenomas of the colon (supplementary material, Figure S5C) and in adenocarcinoma (supplementary material, Figure S5D). Subsequent approval was obtained from the NHS Greater Glasgow and Clyde Biorepository for access to two Glasgow Royal Infirmary Academic Unit of Surgery colorectal TMAs. The first (screen‐detected TMA) contains cores from 182 patients (three cores per tumour) who had undergone resection of early T1/T2 polyp cancers identified via the NHS Bowel Screening Programme, while the second (non‐screen‐detected TMA) contains cores from 272 patients (four cores per tumour) who underwent surgery for stage I–III CRC between 1997 and 2005.

The patient demographics and clinicopathological features of the tumours are summarised in the supplementary material, Table S4. The TMA was stained for N‐WASP and scored using a weighted histoscore method (supplementary material, Figure S6A). Patients were divided into groups according to their N‐WASP histoscores relative to the median. Chi‐squared analysis revealed a correlation between N‐WASP expression and age under 65 years at diagnosis (*p =* 0.007), which may reflect genetic differences in CRCs presenting in younger patients (supplementary material, Table S5). As screening starts from age 50 in Scotland, our cohort had an age range of 50–76 years and a mean age of 65.72 years. Poorly differentiated tumours (*n* = 4) all expressed high levels of N‐WASP (*p =* 0.044). As this TMA was only recently constructed, survival data are not available as most patients are still living.

### N‐WASP is correlated with MMR (DNA mismatch repair) status but not with survival in human colorectal cancers

**Figure 6:**
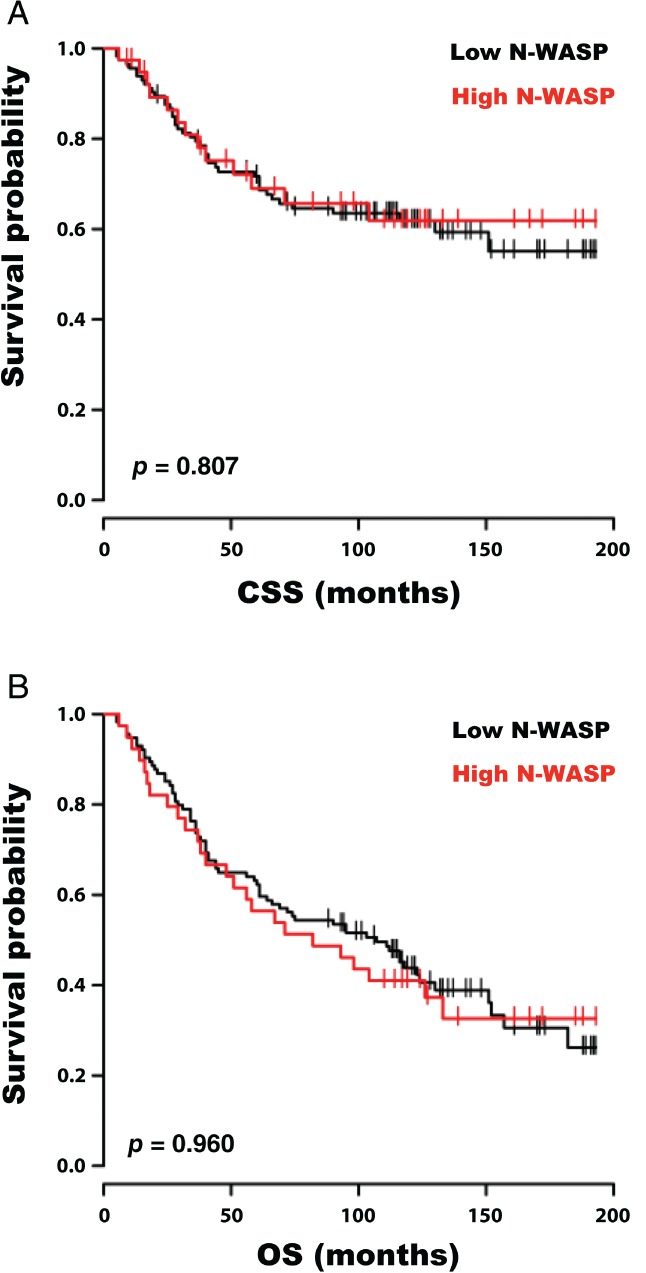
N‐WASP in human colorectal cancer – TMA. (A) Cancer‐specific survival curves (months since surgery) and (B) overall survival curves for patients with tumours with high (median and above, red line, n = 75) and low (below median, black line, n = 78) epithelial IHC histoscores for N‐WASP. P value derived from log‐rank test.

N‐WASP was analysed in the non‐screen‐detected TMA – a cohort of 272 colorectal cancer patients who underwent surgical resection between 1997 and 2005 (stage I–III cancers). The patient demographics and clinicopathological features of the tumours included in the cohort are summarised in the supplementary material, Table S6. The correlation between N‐WASP and a number of clinicopathological characteristics was again compared by chi‐squared analysis (supplementary material, Table S7). The only statistically significant correlation was between low N‐WASP histoscore and MMR deficiency. Patients were again divided into groups according to their N‐WASP histoscores. There was no difference in cancer‐specific (Figure 6A) or overall (Figure 6B) survival between patients with high and low histoscores. When patients were stratified by Dukes stage, there was a divergence of the curves (supplementary material, Figure S6B), with a trend towards better cancer‐specific survival in Dukes B patients with the highest histoscores; however, there were only 70 patients and seven events in this subgroup, and the results were not statistically significant (*p =* 0.118). We therefore cautiously suggest that further investigation of N‐WASP expression in a larger clinical cohort is warranted.

## Discussion

Although expressed at moderate levels throughout normal intestinal epithelium, N‐WASP knockout in intestinal epithelial cells gave a surprisingly mild phenotype, with no obvious morphological changes. Loss of N‐WASP resulted in a slight increase in the number of Paneth and goblet cells and further displacement of their position along the crypt‐villus axis than that caused by *Apc* loss alone. Loss of N‐WASP also increased the distance that cells migrated up along the crypt–villus axis over 24 h. Migration up the crypt–villus axis is thought to be driven mainly by proliferation 30, so this result was surprising as loss of N‐WASP did not alter proliferation. Previous studies found differential expression of integrins and matrix components in crypt–villus regions, which might impact on migration through differential adhesion 36, 37. N‐WASP is also required for matrix remodelling in cancer cells 6, 7, so might be important for epithelial cell interactions with the basement membrane. N‐WASP deletion provides perhaps the first demonstration of a case where migration of intestinal epithelial cells is impacted without alteration of proliferation rates and suggests that the cytoskeleton may be important in crypt–villus migration.

The Paneth cell is a source of Wnt in the intestine and maintains a niche for Lgr5^+^ stem cells 32, 33 as well as secreting antimicrobial compounds to protect against pathogens and maintain the barrier function 38. Although Paneth cell numbers were only slightly increased in *N‐wasp* knockouts, this might be significant as the number and location of Paneth cells are tightly regulated in normal epithelium 39, 40, 41. Furthermore, Paneth cells and goblet cells develop from a common precursor 42, suggesting why both might increase. N‐WASP regulates differentiation and maturation of lymphocytes 27, 43, pancreatic β cells 44, and hair follicle progenitor cells 8. The data presented here suggest that it may have a similar role in cell fate in the intestinal epithelium.

In the *Apc* ^fl/+^ model, *N‐wasp* knockout increased tumour burden and decreased survival. *N‐wasp* knockout *Apc* ^fl/+^ animals developed multiple microadenomas, as well as a few larger lesions (supplementary material, Figure S4). Increased Paneth cell numbers might thus support more tumour‐originating cells 45. However, preliminary studies using RNA *in situ* hybridisation did not show any N‐WASP‐dependent difference in the location of Lgr5^+^ stem cells within small intestinal villi in any of our models (data not shown).

Although *N‐wasp* knockouts harboured more small lesions, there was no difference in the size of the largest individual tumour or proliferation rate. As mice generally reach the endpoint due to a large tumour causing obstruction of the bowel lumen, this suggests that the difference in survival between the two cohorts is due to earlier initiation of tumourigenesis in *N‐wasp* knockouts. *Apc* loss alone will increase the proliferative ability of intestinal epithelial cells but further mutations are required in order for tumours to progress to cancer. Therefore, N‐WASP may have a tumour suppressive role in early lesions.

Although there is very little known about a role for N‐WASP in the GI tract, it stabilises epithelial adherens junctions in cultured cells 46. Competent adherens junctions may pose a mechanical impediment to adenoma formation in *Apc‐*deficient tissue, suggesting that destabilisation could promote progression 46. However, we did not detect any significant changes in architecture or E‐cadherin staining (not shown) of normal or tumour tissue when *N‐wasp* was deleted. No difference in survival or intestinal tumour burden was observed between *Apc* ^*fl/+*^ *KRas* ^G12D/+^ and *Apc* ^*fl/+*^ *KRas* ^G12D/+^ *N‐wasp* ^fl/fl^ mice. Previous research has shown that oncogenic *Ras* causes a reduction in adherens junction formation, lending further support to the proposed model 47.

In the early cancer TMA, all of the poorly differentiated tumours showed a high N‐WASP histoscore. There was also a possible correlation between N‐WASP histoscore and T stage but this did not reach statistical significance. Returning to this cohort at a later time could provide more samples and help to assess the use of N‐WASP histoscore as a prognostic indicator for early cancers. Within the advanced cancer TMA cohort, the only significant correlation was that MMR‐deficient tumours had a greater frequency of low than of high N‐WASP histoscores. There was no difference in overall or cancer‐specific survival between patients with high or low histoscores. However, subgroup analysis revealed poorer survival in patients with Dukes stage A tumours and high epithelial N‐WASP histoscores and Dukes B tumours with low histoscores, although the differences were not statistically significant. The lack of statistical significance, however, may be related to the small sample size, as there were only 14 Dukes A patients and 70 Dukes B patients, so the results do not definitively exclude N‐WASP as a prognostic indicator. Indeed, the findings from the database searches of Oncomine and cBioPortal show decreased expression of both *APC* and *N‐WASP* in colorectal adenomas and carcinomas, and that lower *N‐WASP* expression is associated with poorer survival. The clinicopathological data available via such methods, however, are very limited and it would be worthwhile to explore N‐WASP in a larger TMA cohort to explore its usefulness as a prognostic biomarker in clinical practice.

## Author contributions statement

HTM and LMM conceptualised the study. HTM, LMM, OJS, and FAC were responsible for methodology. HTM, LF, HJS, and RP conducted investigations. HTM, DFV, and RP analysed the data. HTM and LMM acquired funding. LMM, OJS, JP, and SBS contributed essential reagents. LMM supervised the study. All authors were involved in writing the paper and had final approval of the submitted and published versions.

**SUPPLEMENTARY MATERIAL ONLINE**

**Supplementary figure legends**

**Figure S1.** N‐WASP knockout in colon at 4 days and 1 year and in intestine at 1 year

**Figure S2.** Effect of N‐WASP knockout on colonic epithelial proliferation and differentiation of intestinal specialized cell types and apoptosis in a rapid (3–4 day) model

**Figure S3.** Effect of N‐WASP knockout on tumour burden in *Apc* and *Kras* models of intestinal tumourigenesis

**Figure S4.** Representative images of microadenomas and adenomas

**Figure S5.** N‐WASP in human intestine, colon, adenomas, and adenocarcinomas

**Figure S6.** TMA scoring

**Table S1.** N‐WASP knockout cohorts

**Table S2.** Intestinal turnover cohorts

**Table S3.** Tumour cohorts

**Table S4.** Clinicopathological characteristics of the screen‐detected cancer cohort

**Table S5.** Correlation of N‐WASP histoscore and clinicopathological characteristics in the screen‐detected cancer TMA

**Table S6.** Clinicopathological characteristics of the non‐screen‐detected cancer cohort

**Table S7.** Correlation of N‐WASP histoscore and clinicopathological characteristics in the non‐screen‐detected cancer TMA

## Supporting information


**Supplementary figure legends**

**Figure S1.** N‐WASP knockout in colon at 4 days and 1 year and in intestine at 1 year. (A) Position of the lowest BrdU‐positive cell (measured as distance from crypt base) in wild‐type (WT) and N‐wasp ^fl/fl^ (KO) intestine at 4 days (black) and 1 year (red) post‐tamoxifen induction. n = 3–5. Error bars = SEM. (B) Number of BrdU‐positive cells per half crypt/villus unit in wild‐type (WT) and N‐wasp ^fl/fl^ (KO) colon at 4 days (black) and 1 year (red) post‐tamoxifen induction. n = 3–5. Error bars = SEM. ^†^Crypt = half crypt/villus unit. (C) Colon crypt height (measured as distance of highest BrdU‐positive cell from crypt base) in wild‐type (WT) and N‐wasp ^fl/fl^ (KO) colon at 4 days (black) and 1 year (red) post‐tamoxifen induction. n = 3–5. Error bars = SEM. (D) Number of apoptotic cells [measured by cleaved caspase 3 (CC3) positivity] per 100 half crypt/villus units in wild‐type (WT) and N‐wasp ^fl/fl^ (KO) intestine at 4 days (black) and 1 year (red) post‐tamoxifen induction. n = 5–6. ^†^Crypt = half crypt/villus unit. (E) Representative images of N‐WASP IHC in N‐wasp ^fl/fl^ intestine (top row) and colon (bottom row) 1 year post‐tamoxifen induction. White scale bars = 500 μm; black scale bars = 100 μm. (F) Cell migration along the intestinal crypt–villus axis as assessed by change in position of the highest BrdU‐positive cell at 2 h (black) and 24 h (red) in WT and KO intestines, 4 days post‐tamoxifen induction. n = 4–5. Error bars = SEM. *p < 0.05; ***p < 0.001 (Mann–Whitney). (G) Cell migration along the intestinal crypt–villus axis as assessed by change in position of the highest BrdU‐positive cell at 2 h (black) and 24 h (red) in WT and KO colons, 1 year post‐tamoxifen induction. n = 3. Error bars = SEM. **p < 0.01 (Mann–Whitney).

**Figure S2.** Effect of N‐wasp knockout on colonic epithelial proliferation and differentiation of intestinal specialized cell types and apoptosis in a rapid (3–4 day) model. (A) Number of BrdU‐positive cells per half crypt in wild‐type (WT), Apc ^fl/fl^ (A), Apc ^fl/fl^ N‐wasp ^fl/fl^ (AN), Apc ^fl/fl^ Kras ^G12D/+^ (AK), and Apc ^fl/fl^ Kras ^G12D/+^ N‐wasp ^fl/fl^ (AKN) colons. (B) Position of the highest BrdU‐positive cell (measured as distance form crypt base) in wild‐type (WT), Apc ^fl/fl^ (A), Apc ^fl/fl^ N‐wasp ^fl/fl^ (AN), Apc ^fl/fl^ KRAS ^G12D/+^ (AK), and Apc ^fl/fl^ Kras ^G12D/+^ N‐wasp ^fl/fl^ (AKN) intestines.(C) Representative images of special stain ABPAS to identify goblet cells and number of goblet cells (GCs) per half crypt/villus unit in wild‐type (WT), Apc ^fl/fl^ (A), Apc ^fl/fl^ N‐wasp ^fl/fl^ (AN), Apc ^fl/fl^ Kras ^G12D/+^ (AK), and Apc ^fl/fl^ Kras ^G12D/+^ N‐wasp ^fl/fl^ (AKN) intestines. (D) Number of enteroendocrine cells (EECs) per 100 half crypt/villus units in wild‐type (WT), Apc ^fl/fl^ (A), Apc ^fl/fl^ N‐wasp ^fl/fl^ (AN), Apc ^fl/fl^ Kras ^G12D/+^ (AK), and Apc ^fl/fl^ Kras ^G12D/+^ N‐wasp ^fl/fl^ (AKN) intestines. (E) Number of apoptotic cells [measured by cleaved caspase 3 (CC3) positivity] per 100 half crypt/villus units in wild‐type (WT), Apc ^fl/fl^ (A), Apc ^fl/fl^ N‐wasp ^fl/fl^ (AN), Apc ^fl/fl^ Kras ^G12D/+^ (AK), and Apc ^fl/fl^ Kras ^G12D/+^ N‐wasp ^fl/fl^ (AKN) intestines. All graphs: n = 4–5. Error bars = SEM. ns = not significant; *p < 0.05 (Mann–Whitney). ^†^Crypt = half crypt/villus unit.

**Figure S3.** Effect of N‐wasp knockout on tumour burden in Apc and Kras models of intestinal tumourigenesis. (A) Total colonic tumour count in Apc ^fl/+^ (A, n = 13) and Apc ^fl/+^ N‐wasp ^fl/fl^ (AN, n = 15) mice. (B) Average colonic tumour area in A and AN mice (n = 4). (C) Ki67 positivity in A and AN intestinal tumours (n = 5). (D) Total intestinal tumour count in Apc ^fl/+^ Kras ^G12D/+^ (AK, n = 9) and Apc ^fl/+^ Kras ^G12D/+^ N‐wasp ^fl/fl^ (AKN, n = 11) mice. (E) Average intestinal tumour area in AK and AKN mice (n = 5). (F) Total number of colonic tumours in AK (n = 9) and AKN (n = 11) mice. (G) Average colonic tumour area in A and AKN mice (n = 5). All graphs: error bars = SEM. *p < 0.05; **p < 0.01 (Mann–Whitney).

**Figure S4.** (A) Representative images of microadenomas in Apc ^fl/+^ ‘A' (top panel) and Apc ^fl/+^ N‐wasp ^fl/fl^ (AN) (bottom panel) intestinal tumours. Scale bars = 100 μm. (B) Representative images of adenomas in Apc ^fl/+^ (A) (top panel) and Apc ^fl/+^ N‐wasp ^fl/fl^ (AN) (bottom panel) colonic tumours. Scale bars = 100 μm.

**Figure S5.** N‐WASP in human intestine, colon, adenomas, and adenocarcinomas. (A) Representative image of normal human small intestine stained by IHC for N‐WASP (left panel, with zoom of black box right panel). (B) Representative image of normal human colon stained by IHC for N‐WASP (left panel, with zoom of black box right panel). (C) Representative image of human colonic adenoma stained by IHC for N‐WASP, with zoom of black box right panel. (D) Representative image of human colorectal cancer stained by IHC for N‐WASP, showing both tumour surface (left panel) and the invasive front (right panel). Yellow scale bars = 100 μm; black scale bars = 250 μm; white scale bars = 1000 μm.

**Figure S6.** TMA scoring. (A) Representative images of TMA cores scored weakly (left panel), moderately (centre panel) or strongly (right panel) positive for N‐WASP protein expression as assessed by IHC. Scale bars = 250 μm. (B) Cancer‐specific survival curves (months since surgery) for patients with tumours with high (third quartile and above, red line, n = 20) and low (below third quartile, black line, n = 50) epithelial IHC histoscores for N‐WASP. P value derived from the log‐rank test.

**Table S1**. N‐wasp knockout cohorts
**Table S2**. Intestinal turnover cohorts
**Table S3**. Tumour cohorts
**Table S4**. Clinicopathological characteristics of the screen‐detected cancer cohort
**Table S5**. Correlation of N‐WASP histoscore and clinicopathological characteristics in the screen‐detected cancer TMA. P values derived from chi‐squared test, n = 159
**Table S6**. Clinicopathological characteristics of the non‐screen‐detected cancer cohort
**Table S7**. Correlation of N‐WASP histoscore and clinicopathological characteristics in the non‐screen‐detected cancer TMA. P values derived from chi‐squared test, n = 153
