# Supplementary material for: Loss of N‐WASP drives early progression in an Apc model of intestinal tumourigenesis
Source: J Pathol. 2018 May 28;245(3):337–48. doi: 10.1002/path.5086 (PMC6033012; doi:10.1002/path.5086)
Supplement: Supplementary file 1 — Supplementary figure legends [file PATH-245-337-s001.docx]

**Supplementary figure legends**

**Figure S1.** N-WASP knockout in colon at 4 days and 1 year and in intestine at 1 year. (A) Position of the lowest BrdU-positive cell (measured as distance from crypt base) in wild-type (WT) and *N-wasp*^fl/fl^ (KO) intestine at 4 days (black) and 1 year (red) post-tamoxifen induction. *n =* 3–5. Error bars = SEM. (B) Number of BrdU-positive cells per half crypt/villus unit in wild-type (WT) and *N-wasp* ^fl/fl^ (KO) colon at 4 days (black) and 1 year (red) post-tamoxifen induction. *n =* 3–5. Error bars = SEM. ^†^Crypt = half crypt/villus unit. (C) Colon crypt height (measured as distance of highest BrdU-positive cell from crypt base) in wild-type (WT) and *N-wasp*^fl/fl^ (KO) colon at 4 days (black) and 1 year (red) post-tamoxifen induction. *n =* 3–5. Error bars = SEM. (D) Number of apoptotic cells [measured by cleaved caspase 3 (CC3) positivity] per 100 half crypt/villus units in wild-type (WT) and *N-wasp*^fl/fl^ (KO) intestine at 4 days (black) and 1 year (red) post-tamoxifen induction. *n =* 5–6. ^†^Crypt = half crypt/villus unit. (E) Representative images of N-WASP IHC in *N-wasp*^fl/fl^ intestine (top row) and colon (bottom row) 1 year post-tamoxifen induction. White scale bars = 500 μm; black scale bars = 100 μm. (F) Cell migration along the intestinal crypt–villus axis as assessed by change in position of the highest BrdU-positive cell at 2 h (black) and 24 h (red) in WT and KO intestines, 4 days post-tamoxifen induction. *n =* 4–5. Error bars = SEM. **p* < 0.05; ****p* < 0.001 (Mann–Whitney). (G) Cell migration along the intestinal crypt–villus axis as assessed by change in position of the highest BrdU-positive cell at 2 h (black) and 24 h (red) in WT and KO colons, 1 year post-tamoxifen induction. *n =* 3. Error bars = SEM. ***p* < 0.01 (Mann–Whitney).

**Figure S2.** Effect of *N-wasp* knockout on colonic epithelial proliferation and differentiation of intestinal specialized cell types and apoptosis in a rapid (3–4 day) model. (A) Number of BrdU-positive cells per half crypt in wild-type (WT), *Apc*^fl/fl^ (A), *Apc*^fl/fl^*N-wasp*^fl/fl^ (AN), *Apc*^fl/fl^*Kras*^G12D/+^ (AK), and *Apc*^fl/fl^*Kras*^G12D/+^*N-wasp*^fl/fl^ (AKN) colons. (B) Position of the highest BrdU-positive cell (measured as distance form crypt base) in wild-type (WT), *Apc*^fl/fl^ (A), *Apc*^fl/fl^*N-wasp*^fl/fl^ (AN), *Apc*^fl/fl^*KRAS*^G12D/+^ (AK), and *Apc*^fl/fl^*Kras*^G12D/+^*N-wasp*^fl/fl^ (AKN) intestines.(C) Representative images of special stain ABPAS to identify goblet cells and number of goblet cells (GCs) per half crypt/villus unit in wild-type (WT), *Apc*^fl/fl^ (A), *Apc*^fl/fl^*N-wasp*^fl/fl^ (AN), *Apc*^fl/fl^*Kras*^G12D/+^ (AK), and *Apc*^fl/fl^*Kras*^G12D/+^*N-wasp*^fl/fl^ (AKN) intestines. (D) Number of enteroendocrine cells (EECs) per 100 half crypt/villus units in wild-type (WT), *Apc*^fl/fl^ (A), *Apc*^fl/fl^*N-wasp*^fl/fl^ (AN), *Apc*^fl/fl^*Kras*^G12D/+^ (AK), and *Apc*^fl/fl^*Kras*^G12D/+^*N-wasp*^fl/fl^ (AKN) intestines. (E) Number of apoptotic cells [measured by cleaved caspase 3 (CC3) positivity] per 100 half crypt/villus units in wild-type (WT), *Apc*^fl/fl^ (A), *Apc*^fl/fl^*N-wasp*^fl/fl^ (AN), *Apc*^fl/fl^*Kras*^G12D/+^ (AK), and *Apc*^fl/fl^*Kras*^G12D/+^*N-wasp*^fl/fl^ (AKN) intestines. All graphs: *n =* 4–5. Error bars = SEM. ns = not significant; **p* < 0.05 (Mann–Whitney). ^†^Crypt = half crypt/villus unit.

**Figure S3.** Effect of *N-wasp* knockout on tumour burden in *Apc* and *Kras* models of intestinal tumourigenesis. (A) Total colonic tumour count in *Apc*^fl/+^ (A, *n =* 13) and *Apc*^fl/+^*N-wasp*^fl/fl^ (AN, *n =* 15) mice. (B) Average colonic tumour area in A and AN mice (*n =* 4). (C) Ki67 positivity in A and AN intestinal tumours (*n =* 5). (D) Total intestinal tumour count in *Apc*^fl/+^ *Kras*^G12D/+^ (AK, *n =* 9) and *Apc*^fl/+^ *Kras*^G12D/+^ *N-wasp*^fl/fl^ (AKN, *n =* 11) mice. (E) Average intestinal tumour area in AK and AKN mice (*n =* 5). (F) Total number of colonic tumours in AK (*n =* 9) and AKN (*n =* 11) mice. (G) Average colonic tumour area in A and AKN mice (*n =* 5). All graphs: error bars = SEM. **p* < 0.05; ***p* < 0.01 (Mann–Whitney).

**Figure S4.** (A) Representative images of microadenomas in *Apc*^fl/+^ ‘A’ (top panel) and *Apc*^fl/+^*N-wasp*^fl/fl^ (AN) (bottom panel) intestinal tumours. Scale bars = 100 μm. (B) Representative images of adenomas in *Apc*^fl/+^ (A) (top panel) and *Apc*^fl/+^*N-wasp*^fl/fl^ (AN) (bottom panel) colonic tumours. Scale bars = 100 μm.

**Figure S5.** N-WASP in human intestine, colon, adenomas, and adenocarcinomas. (A) Representative image of normal human small intestine stained by IHC for N-WASP (left panel, with zoom of black box right panel). (B) Representative image of normal human colon stained by IHC for N-WASP (left panel, with zoom of black box right panel). (C) Representative image of human colonic adenoma stained by IHC for N-WASP, with zoom of black box right panel. (D) Representative image of human colorectal cancer stained by IHC for N-WASP, showing both tumour surface (left panel) and the invasive front (right panel). Yellow scale bars = 100 μm; black scale bars = 250 μm; white scale bars = 1000 μm.

**Figure S6.** TMA scoring. (A) Representative images of TMA cores scored weakly (left panel), moderately (centre panel) or strongly (right panel) positive for N-WASP protein expression as assessed by IHC. Scale bars = 250 μm. (B) Cancer-specific survival curves (months since surgery) for patients with tumours with high (third quartile and above, red line, *n* = 20) and low (below third quartile, black line, *n* = 50) epithelial IHC histoscores for N-WASP. *P* value derived from the log-rank test.
