# Supplementary figures and images for: Loss of N‐WASP drives early progression in an Apc model of intestinal tumourigenesis
Source: J Pathol. 2018 May 28;245(3):337–48. doi: 10.1002/path.5086 (PMC6033012; doi:10.1002/path.5086)

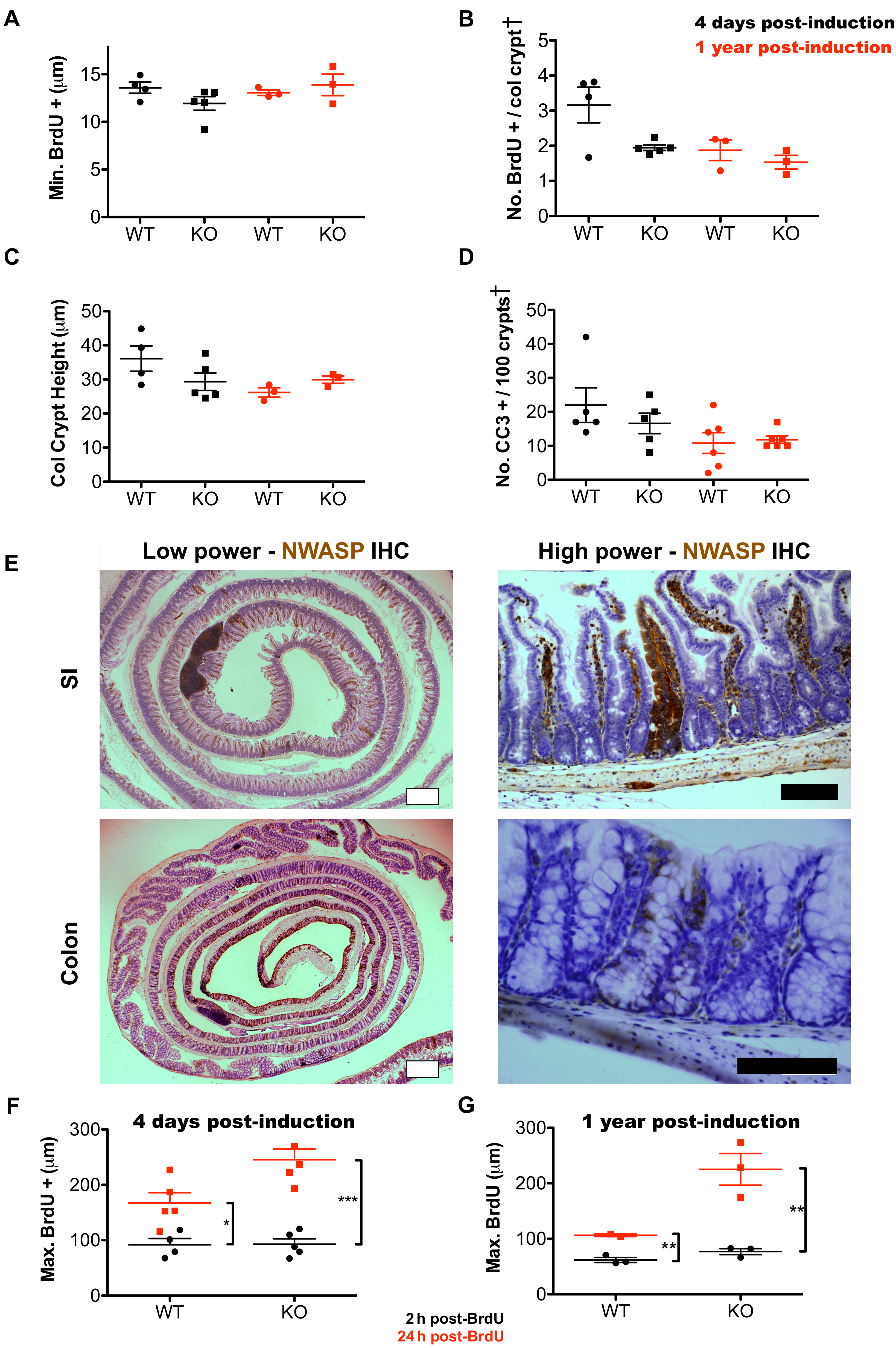

Supplement: Supplementary file 2 — Figure S1. N‐WASP knockout in colon at 4 days and 1 year and in intestine at 1 year. (A) Position of the lowest BrdU‐positive cell (measured as distance from crypt base) in wild‐type (WT) and N‐wasp fl/fl (KO) intestine at 4 days (black) and 1 year (red) post‐tamoxifen induction. n = 3–5. Error bars = SEM. (B) Number of BrdU‐positive cells per half crypt/villus unit in wild‐type (WT) and N‐wasp fl/fl (KO) colon at 4 days (black) and 1 year (red) post‐tamoxifen induction. n = 3–5. Error bars = SEM. †Crypt = half crypt/villus unit. (C) Colon crypt height (measured as distance of highest BrdU‐positive cell from crypt base) in wild‐type (WT) and N‐wasp fl/fl (KO) colon at 4 days (black) and 1 year (red) post‐tamoxifen induction. n = 3–5. Error bars = SEM. (D) Number of apoptotic cells [measured by cleaved caspase 3 (CC3) positivity] per 100 half crypt/villus units in wild‐type (WT) and N‐wasp fl/fl (KO) intestine at 4 days (black) and 1 year (red) post‐tamoxifen induction. n = 5–6. †Crypt = half crypt/villus unit. (E) Representative images of N‐WASP IHC in N‐wasp fl/fl intestine (top row) and colon (bottom row) 1 year post‐tamoxifen induction. White scale bars = 500 μm; black scale bars = 100 μm. (F) Cell migration along the intestinal crypt–villus axis as assessed by change in position of the highest BrdU‐positive cell at 2 h (black) and 24 h (red) in WT and KO intestines, 4 days post‐tamoxifen induction. n = 4–5. Error bars = SEM. *p < 0.05; ***p < 0.001 (Mann–Whitney). (G) Cell migration along the intestinal crypt–villus axis as assessed by change in position of the highest BrdU‐positive cell at 2 h (black) and 24 h (red) in WT and KO colons, 1 year post‐tamoxifen induction. n = 3. Error bars = SEM. **p < 0.01 (Mann–Whitney). [file PATH-245-337-s002.tif]

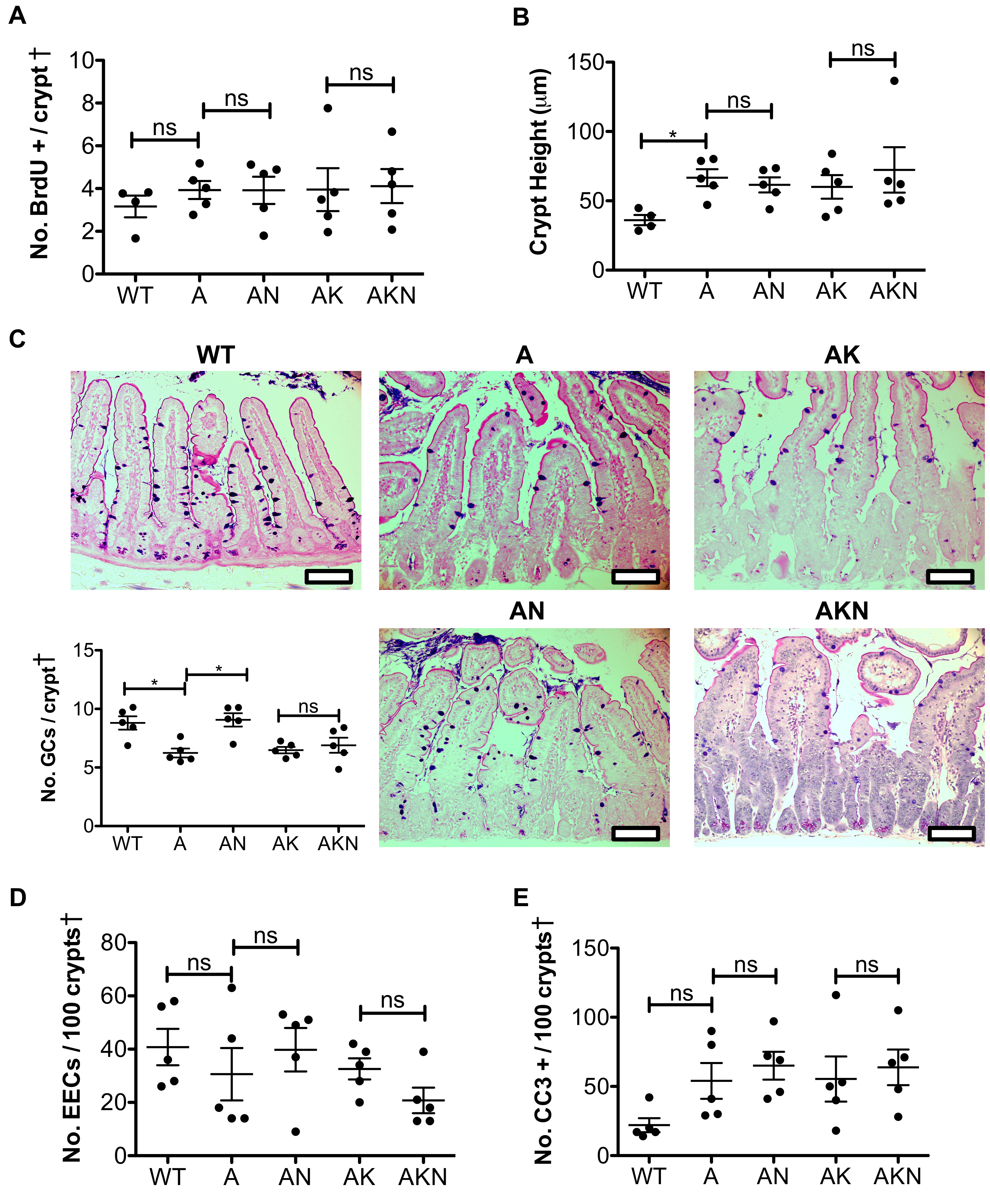

Supplement: Supplementary file 3 — Figure S2. Effect of N‐wasp knockout on colonic epithelial proliferation and differentiation of intestinal specialized cell types and apoptosis in a rapid (3–4 day) model. (A) Number of BrdU‐positive cells per half crypt in wild‐type (WT), Apc fl/fl (A), Apc fl/fl N‐wasp fl/fl (AN), Apc fl/fl Kras G12D/+ (AK), and Apc fl/fl Kras G12D/+ N‐wasp fl/fl (AKN) colons. (B) Position of the highest BrdU‐positive cell (measured as distance form crypt base) in wild‐type (WT), Apc fl/fl (A), Apc fl/fl N‐wasp fl/fl (AN), Apc fl/fl KRAS G12D/+ (AK), and Apc fl/fl Kras G12D/+ N‐wasp fl/fl (AKN) intestines.(C) Representative images of special stain ABPAS to identify goblet cells and number of goblet cells (GCs) per half crypt/villus unit in wild‐type (WT), Apc fl/fl (A), Apc fl/fl N‐wasp fl/fl (AN), Apc fl/fl Kras G12D/+ (AK), and Apc fl/fl Kras G12D/+ N‐wasp fl/fl (AKN) intestines. (D) Number of enteroendocrine cells (EECs) per 100 half crypt/villus units in wild‐type (WT), Apc fl/fl (A), Apc fl/fl N‐wasp fl/fl (AN), Apc fl/fl Kras G12D/+ (AK), and Apc fl/fl Kras G12D/+ N‐wasp fl/fl (AKN) intestines. (E) Number of apoptotic cells [measured by cleaved caspase 3 (CC3) positivity] per 100 half crypt/villus units in wild‐type (WT), Apc fl/fl (A), Apc fl/fl N‐wasp fl/fl (AN), Apc fl/fl Kras G12D/+ (AK), and Apc fl/fl Kras G12D/+ N‐wasp fl/fl (AKN) intestines. All graphs: n = 4–5. Error bars = SEM. ns = not significant; *p < 0.05 (Mann–Whitney). †Crypt = half crypt/villus unit. [file PATH-245-337-s003.tif]

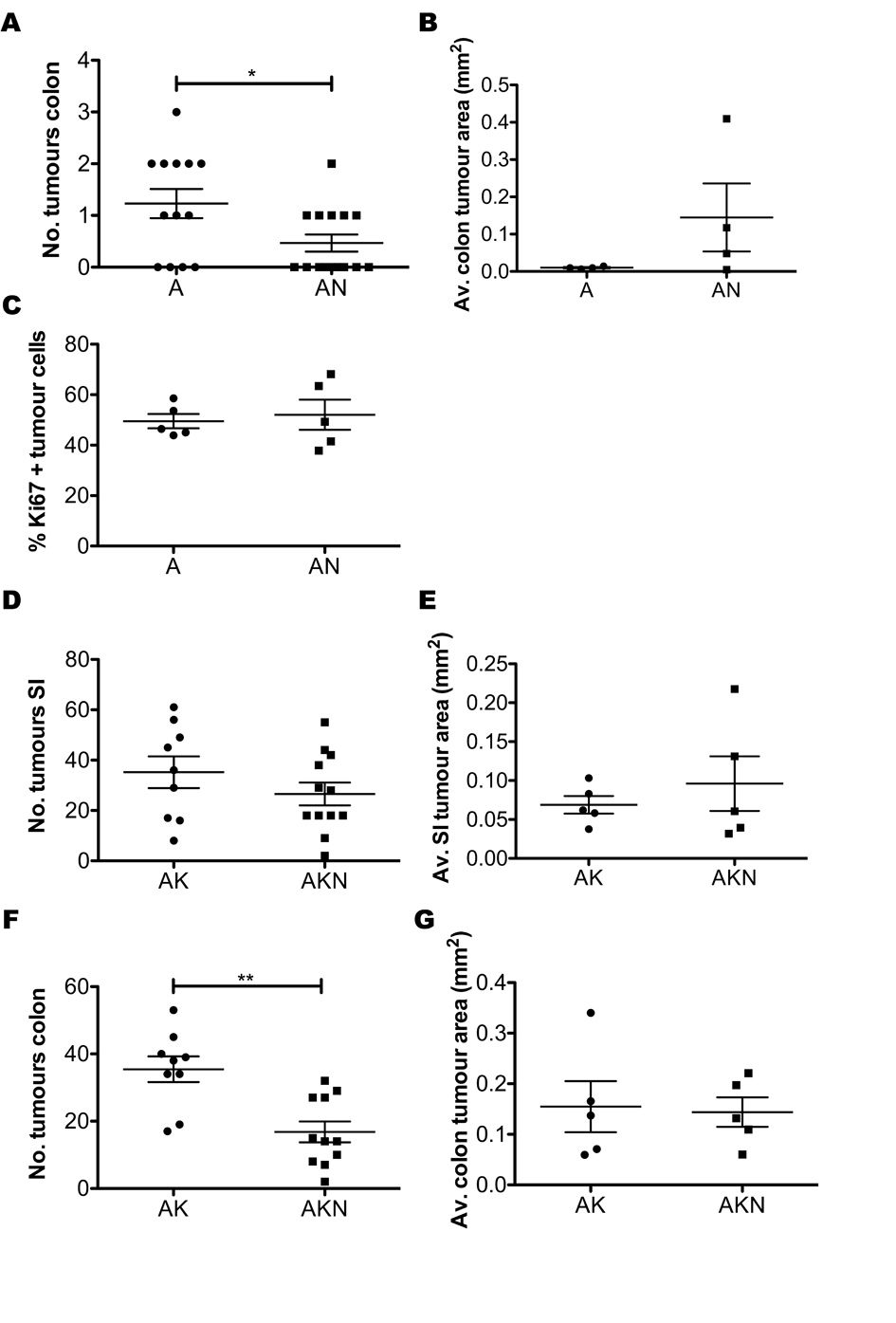

Supplement: Supplementary file 4 — Figure S3. Effect of N‐wasp knockout on tumour burden in Apc and Kras models of intestinal tumourigenesis. (A) Total colonic tumour count in Apc fl/+ (A, n = 13) and Apc fl/+ N‐wasp fl/fl (AN, n = 15) mice. (B) Average colonic tumour area in A and AN mice (n = 4). (C) Ki67 positivity in A and AN intestinal tumours (n = 5). (D) Total intestinal tumour count in Apc fl/+ Kras G12D/+ (AK, n = 9) and Apc fl/+ Kras G12D/+ N‐wasp fl/fl (AKN, n = 11) mice. (E) Average intestinal tumour area in AK and AKN mice (n = 5). (F) Total number of colonic tumours in AK (n = 9) and AKN (n = 11) mice. (G) Average colonic tumour area in A and AKN mice (n = 5). All graphs: error bars = SEM. *p < 0.05; **p < 0.01 (Mann–Whitney). [file PATH-245-337-s004.tif]

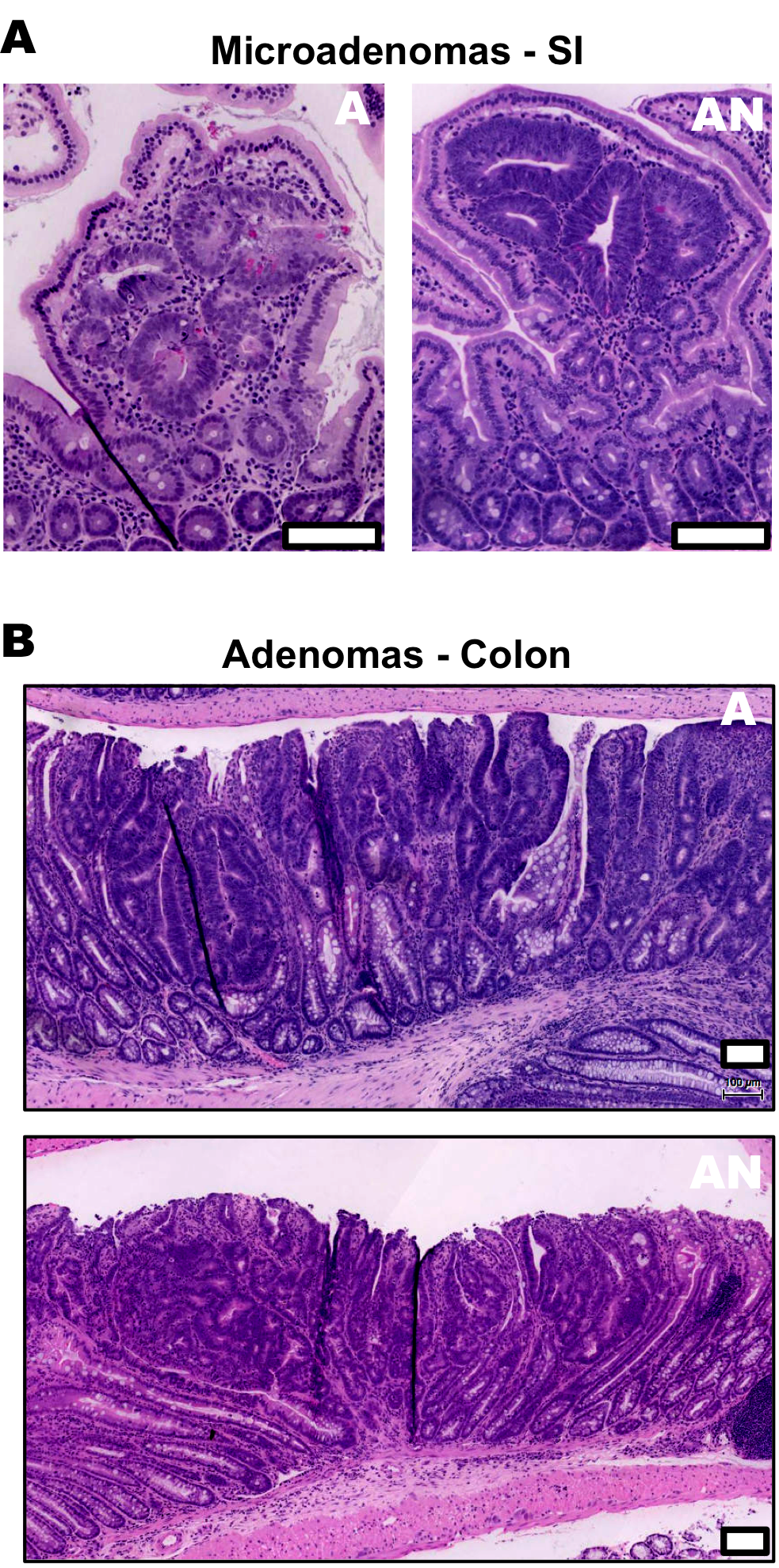

Supplement: Supplementary file 5 — Figure S4. (A) Representative images of microadenomas in Apc fl/+ ‘A' (top panel) and Apc fl/+ N‐wasp fl/fl (AN) (bottom panel) intestinal tumours. Scale bars = 100 μm. (B) Representative images of adenomas in Apc fl/+ (A) (top panel) and Apc fl/+ N‐wasp fl/fl (AN) (bottom panel) colonic tumours. Scale bars = 100 μm. [file PATH-245-337-s005.tiff]

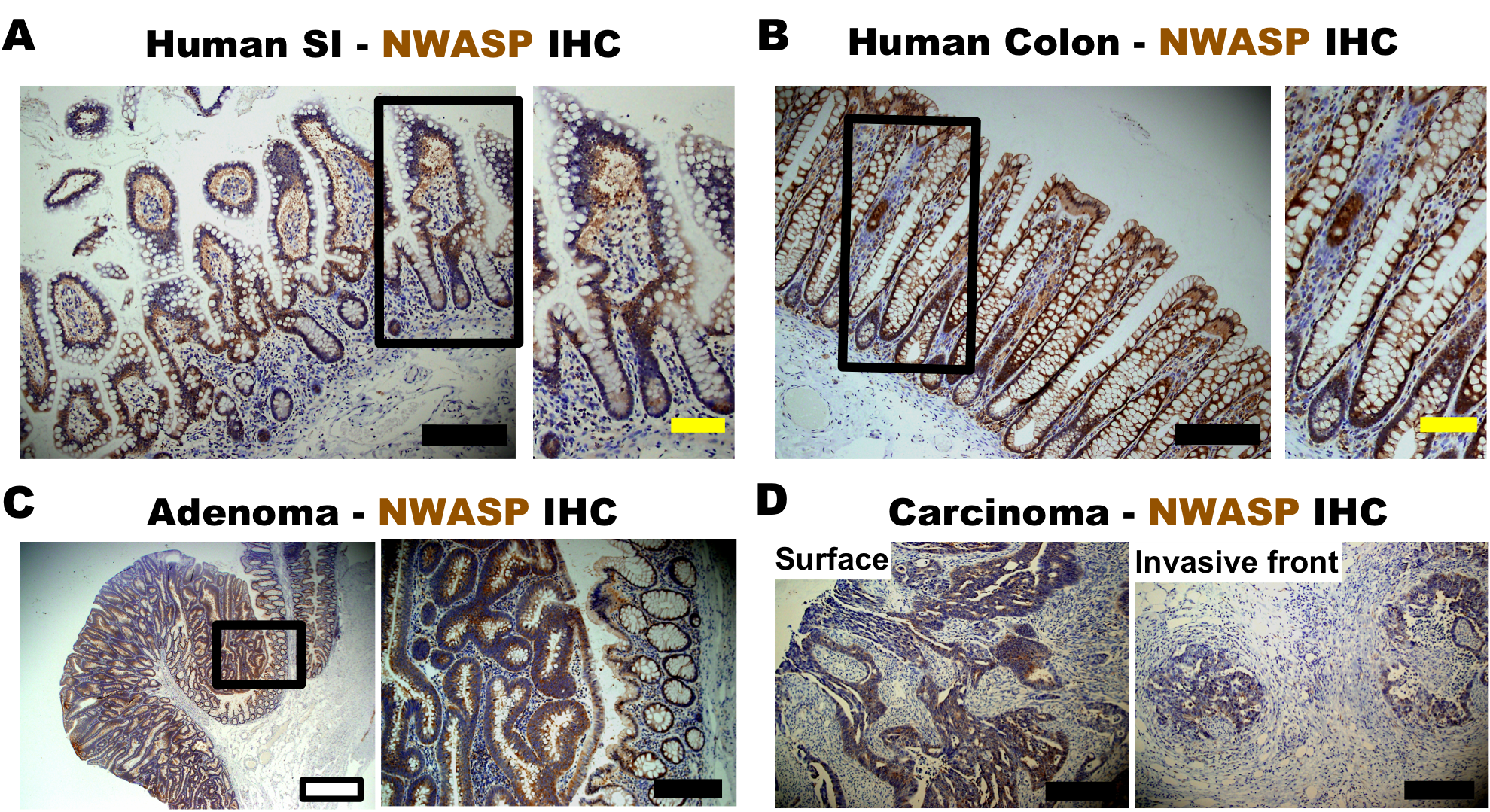

Supplement: Supplementary file 6 — Figure S5. N‐WASP in human intestine, colon, adenomas, and adenocarcinomas. (A) Representative image of normal human small intestine stained by IHC for N‐WASP (left panel, with zoom of black box right panel). (B) Representative image of normal human colon stained by IHC for N‐WASP (left panel, with zoom of black box right panel). (C) Representative image of human colonic adenoma stained by IHC for N‐WASP, with zoom of black box right panel. (D) Representative image of human colorectal cancer stained by IHC for N‐WASP, showing both tumour surface (left panel) and the invasive front (right panel). Yellow scale bars = 100 μm; black scale bars = 250 μm; white scale bars = 1000 μm. [file PATH-245-337-s006.tif]

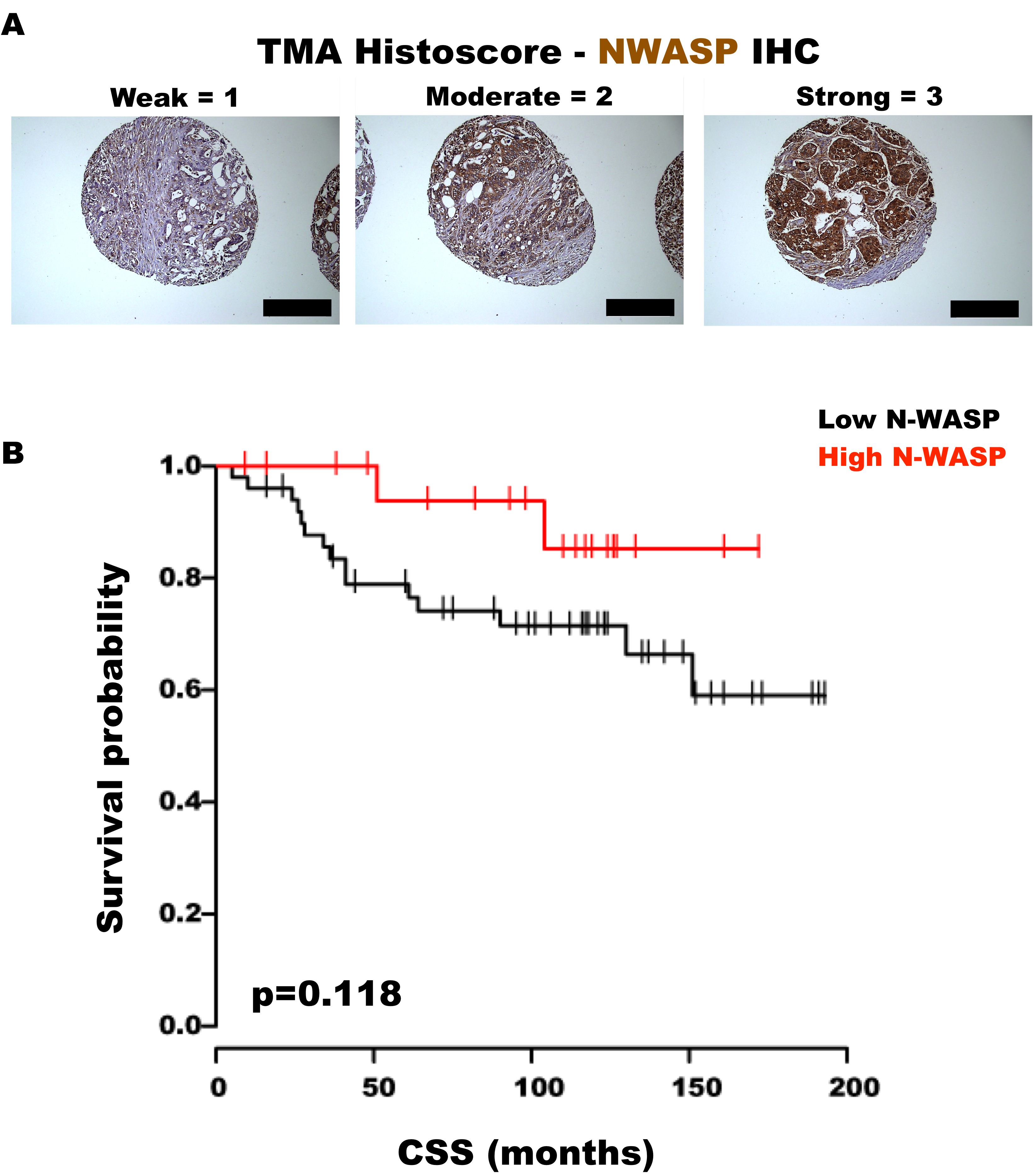

Supplement: Supplementary file 7 — Figure S6. TMA scoring. (A) Representative images of TMA cores scored weakly (left panel), moderately (centre panel) or strongly (right panel) positive for N‐WASP protein expression as assessed by IHC. Scale bars = 250 μm. (B) Cancer‐specific survival curves (months since surgery) for patients with tumours with high (third quartile and above, red line, n = 20) and low (below third quartile, black line, n = 50) epithelial IHC histoscores for N‐WASP. P value derived from the log‐rank test. [file PATH-245-337-s007.tif]
