# Supplementary material for: Loss of N‐WASP drives early progression in an Apc model of intestinal tumourigenesis
Source: J Pathol. 2018 May 28;245(3):337–48. doi: 10.1002/path.5086 (PMC6033012; doi:10.1002/path.5086)
Supplement: Supplementary file 8 — Table S1. N‐wasp knockout cohorts Table S2. Intestinal turnover cohorts Table S3. Tumour cohorts Table S4. Clinicopathological characteristics of the screen‐detected cancer cohort Table S5. Correlation of N‐WASP histoscore and clinicopathological characteristics in the screen‐detected cancer TMA. P values derived from chi‐squared test, n = 159 Table S6. Clinicopathological characteristics of the non‐screen‐detected cancer cohort Table S7. Correlation of N‐WASP histoscore and clinicopathological characteristics in the non‐screen‐detected cancer TMA. P values derived from chi‐squared test, n = 153 [file PATH-245-337-s008.docx]

**Table S1.** *N-wasp* knockout cohorts

| **Cohort** | **ID** | **Sex** | **Vil** | **A** | **K** | **N** | **Days in unit** | **Age at ind (weeks)** | **Birth–cull (days)** | **Ind–cull (days)** |
| --- | --- | --- | --- | --- | --- | --- | --- | --- | --- | --- |
| WT-4d 2h B | 178688 | F | + | wt | wt | wt | 29.00 | 12.43 | 91.00 | 4.00 |
| WT-4d 2h B | 178691 | M | + | wt | wt | wt | 16.00 | 9.86 | 73.00 | 4.00 |
| WT-4d 2h B | 178694 | M | + | wt | wt | wt | 16.00 | 9.86 | 73.00 | 4.00 |
| WT-4d 2h B | 187364 | M | + | wt | wt | wt | 8.00 | 11.00 | 81.00 | 4.00 |
| WT-4d 2h B | 189857 | F | + | wt | wt | wt | 13.00 | 10.00 | 74.00 | 4.00 |
| WT-4d 24h B | 178695 | M | + | wt | wt | wt | 17.00 | 10.00 | 74.00 | 4.00 |
| WT-4d 24h B | 178697 | M | + | wt | wt | wt | 17.00 | 10.00 | 74.00 | 4.00 |
| WT-4d 24h B | 178700 | F | + | wt | wt | wt | 17.00 | 10.29 | 76.00 | 4.00 |
| WT-4d 24h B | 189853 | M | + | wt | wt | wt | 8.00 | 8.71 | 65.00 | 4.00 |
| WT-4d 24h B | 189859 | F | + | wt | wt | wt | 13.00 | 10.00 | 74.00 | 4.00 |
| KO-4d 2h B | 098860 | F | + | wt | wt | hom | 46.00 | 13.29 | 97.00 | 4.00 |
| KO-4d 2h B | 114489 | M | + | wt | wt | hom | 7.00 | 12.29 | 90.00 | 4.00 |
| KO-4d 2h B | 139561 | F | + | wt | wt | hom | 7.00 | 10.57 | 78.00 | 4.00 |
| KO-4d 2h B | 139562 | F | + | wt | wt | hom | 7.00 | 10.57 | 78.00 | 4.00 |
| KO-4d 2h B | 149864 | M | + | wt | wt | hom | 7.00 | 7.57 | 57.00 | 4.00 |
| KO-4d 24h B | 187365 | F | + | wt | wt | hom | 8.00 | 11.00 | 81.00 | 4.00 |
| KO-4d 24h B | 187367 | F | + | wt | wt | hom | 24.00 | 13.86 | 102.00 | 5.00 |
| KO-4d 24h B | 180728 | M | + | wt | wt | hom | 16.00 | 9.43 | 70.00 | 4.00 |
| KO-4d 24h B | 180730 | M | + | wt | wt | hom | 16.00 | 9.43 | 70.00 | 4.00 |
| KO-4d 24h B | 180735 | F | + | wt | wt | hom | 16.00 | 9.43 | 70.00 | 4.00 |
| WT-1y 2h BrdU | 149869 | M | + | wt | wt | hom | nd | nd | 452.00 | nd |
| WT-1y 2h B | 149870 | M | + | wt | wt | hom | nd | nd | 452.00 | nd |
| WT-1y 2h B | 170378 | M | + | wt | wt | wt | nd | nd | 485.00 | nd |
| WT-1y 24h B | 149865 | M | + | wt | wt | het | nd | nd | 452.00 | nd |
| WT-1y 24h B | 149875 | F | + | wt | wt | hom | nd | nd | 452.00 | nd |
| WT-1y 24h B | 170380 | M | + | wt | wt | wt | nd | nd | 485.00 | nd |
| KO-1y 2h B | 124014 | M | + | wt | wt | hom | 5.00 | 13.57 | 562.00 | 467.00 |
| KO-1y 2h B | 124019 | M | + | wt | wt | hom | 5.00 | 13.57 | 562.00 | 467.00 |
| KO-1y 2h B | 128555 | M | + | wt | wt | hom | 5.00 | 10.57 | 542.00 | 468.00 |
| KO-1y 24h B | 124021 | M | + | wt | wt | hom | 5.00 | 13.57 | 562.00 | 467.00 |
| KO-1y 24h B | 124022 | F | + | wt | wt | hom | 5.00 | 13.57 | 562.00 | 467.00 |
| KO-1y 24h B | 128557 | M | + | wt | wt | hom | 5.00 | 10.57 | 542.00 | 468.00 |

Details of mice in *N-wasp* knockout cohorts including identification number (ID), sex, genotype, days in non-barrier unit prior to induction (Days in unit), age in weeks at induction [Age at ind (weeks)], age when culled [Birth–cull (days)], and survival from induction to cull [Ind–cull (days)]. Vil = Villin-Cre^ERT2^; A = APC; K = KRas^G12D^; N = N-wasp; WT = wild type; KO = *N-wasp*^fl/fl^; 4d = 4 days post-induction; 1y = 1 year post-induction; 2h B = 2 h post-BrdU injection; 24h B = 24 h post-BrdU injection; nd = not done.

**Table S2.** Intestinal turnover cohorts

| **Cohort** | **ID** | **Sex** | **Vil** | **A** | **K** | **N** | **Days in unit** | **Age at ind (weeks)** | **Birth–cull (days)** | **Ind–cull (days)** |
| --- | --- | --- | --- | --- | --- | --- | --- | --- | --- | --- |
| WT 2h B | 178688 | F | + | wt | wt | wt | 29.00 | 12.43 | 91.00 | 4.00 |
| WT 2h B | 178691 | M | + | wt | wt | wt | 16.00 | 9.86 | 73.00 | 4.00 |
| WT 2h B | 178694 | M | + | wt | wt | wt | 16.00 | 9.86 | 73.00 | 4.00 |
| WT 2h B | 187364 | M | + | wt | wt | wt | 8.00 | 11.00 | 81.00 | 4.00 |
| WT 2h B | 189857 | F | + | wt | wt | wt | 13.00 | 10.00 | 74.00 | 4.00 |
| WT 24h B | 178695 | M | + | wt | wt | wt | 17.00 | 10.00 | 74.00 | 4.00 |
| WT 24h B | 178697 | M | + | wt | wt | wt | 17.00 | 10.00 | 74.00 | 4.00 |
| WT 24h B | 178700 | F | + | wt | wt | wt | 17.00 | 10.29 | 76.00 | 4.00 |
| WT 24h B | 189853 | M | + | wt | wt | wt | 5.00 | 8.71 | 65.00 | 4.00 |
| WT 24h B | 189859 | F | + | wt | wt | wt | 13.00 | 10.00 | 74.00 | 4.00 |
| A 2h B | 135665 | F | + | hom | wt | wt | 7.00 | 9.57 | 71.00 | 4.00 |
| A 2h B | 140304 | M | + | hom | wt | wt | 8.00 | 7.71 | 58.00 | 4.00 |
| A 2h B | 140308 | F | + | hom | wt | wt | 7.00 | 10.29 | 76.00 | 4.00 |
| A 2h B | 144695 | F | + | hom | wt | wt | 7.00 | 9.86 | 73.00 | 4.00 |
| A 2h B | 144696 | F | + | hom | wt | wt | 14.00 | 10.86 | 80.00 | 4.00 |
| A 24h B | 149823 | F | + | hom | wt | wt | 11.00 | 12.43 | 91.00 | 4.00 |
| A 24h B | 149824 | F | + | hom | wt | wt | 11.00 | 12.43 | 91.00 | 4.00 |
| A 24h B | 154124 | F | + | hom | wt | wt | 22.00 | 13.57 | 99.00 | 4.00 |
| A 24h B | 154125 | F | + | hom | wt | wt | 8.00 | 11.57 | 85.00 | 4.00 |
| A 24h B | 161233 | F | + | hom | wt | wt | 22.00 | 10.14 | 75.00 | 4.00 |
| AN 2h B | 099918 | M | + | hom | wt | hom | 51.00 | 13.29 | 97.00 | 4.00 |
| AN 2h B | 161245 | M | + | hom | wt | hom | 8.00 | 8.00 | 60.00 | 4.00 |
| AN 2h B | 161235 | F | + | hom | wt | hom | 22.00 | 10.14 | 75.00 | 4.00 |
| AN 2h B | 140311 | F | + | hom | wt | hom | 21.00 | 14.14 | 103.00 | 4.00 |
| AN 2h B | 154128 | F | + | hom | wt | hom | 8.00 | 11.57 | 85.00 | 4.00 |
| AN 24h B | 171616 | M | + | hom | wt | hom | 5.00 | 9.29 | 69.00 | 4.00 |
| AN 24h B | 171619 | F | + | hom | wt | hom | 5.00 | 9.29 | 69.00 | 4.00 |
| AN 24h B | 181796 | M | + | hom | wt | hom | 16.00 | 9.00 | 67.00 | 4.00 |
| AN 24h B | 185640 | M | + | hom | wt | hom | 6.00 | 10.57 | 78.00 | 4.00 |
| AN 24h B | 185641 | F | + | hom | wt | hom | 6.00 | 10.57 | 78.00 | 4.00 |
| AK 2h B | 131080 | M | + | hom | het | wt | 6.00 | 11.00 | 80.00 | 3.00 |
| AK 2h B | 135662 | M | + | hom | het | wt | 7.00 | 9.57 | 70.00 | 3.00 |
| AK 2h B | 140312 | F | + | hom | het | wt | 7.00 | 10.29 | 75.00 | 3.00 |
| AK 2h B | 140307 | F | + | hom | het | wt | 7.00 | 10.29 | 75.00 | 3.00 |
| AK 2h B | 147329 | F | + | hom | het | wt | 15.00 | 9.14 | 67.00 | 3.00 |
| AK 24h B | 161246 | M | + | hom | het | wt | 11.00 | 8.43 | 62.00 | 3.00 |
| AK 24h B | 161228 | M | + | hom | het | wt | 25.00 | 10.57 | 77.00 | 3.00 |
| AK 24h B | 161234 | F | + | hom | het | wt | 23.00 | 10.29 | 75.00 | 3.00 |
| AK 24h B | 172013 | M | + | hom | het | wt | 5.00 | 8.86 | 65.00 | 3.00 |
| AK 24h B | 181795 | M | + | hom | het | wt | 13.00 | 8.57 | 63.00 | 3.00 |
| AKN 2h B | 098851 | M | + | hom | het | hom | 42.00 | 13.29 | 96.00 | 3.00 |
| AKN 2h B | 099920 | M | + | hom | het | hom | 42.00 | 13.29 | 96.00 | 3.00 |
| AKN 2h B | 131078 | M | + | hom | het | hom | 6.00 | 11.00 | 80.00 | 3.00 |
| AKN 2h B | 131081 | F | + | hom | het | hom | 6.00 | 11.29 | 82.00 | 3.00 |
| AKN 2h B | 131085 | F | + | hom | het | hom | 6.00 | 11.29 | 82.00 | 3.00 |
| AKN 24h B | 157866 | M | + | hom | het | hom | 11.00 | 9.57 | 70.00 | 3.00 |
| AKN 24h B | 161230 | M | + | hom | het | hom | 11.00 | 8.57 | 63.00 | 3.00 |
| AKN 24h B | 172012 | M | + | hom | het | hom | 24.00 | 11.71 | 85.00 | 3.00 |
| AKN 24h B | 171612 | M | + | hom | het | hom | 5.00 | 9.29 | 68.00 | 3.00 |
| AKN 24h B | 177808 | F | + | hom | het | hom | 13.00 | 11.57 | 84.00 | 3.00 |

Details of mice in intestinal turnover cohorts including identification number (ID), sex, genotype (A = *Apc*, K = *Kras*‑, N = *N-wasp*), days in non-barrier unit prior to induction (Days in unit), age in weeks at induction [Age at ind (weeks)], age when culled [Birth–cull (days)], and survival from induction to cull [Ind–cull (days)]. Vil = Villin-Cre^ERT2^. Cohort type: A = *Apc*^fl/fl^; AN = *Apc*^fl/fl^*N-wasp*^fl/fl^; AK = *Apc*^fl/fl^*Kras*^G12D/+^; AKN = *Apc*^fl/fl^*Kras*^G12D/+^*N-wasp*^fl/fl^. 4d = 4 days post-induction; 1y = 1 year post-induction; 2h B= 2 h post-BrdU injection; 24h B= 24 h post-BrdU injection.

**Table S3.** Tumour cohorts

| **Cohort** | **ID** | **Sex** | **Vil** | **A** | **K** | **N** | **Days in unit** | **Age at ind (weeks)** | **Birth–cull (days)** | **Ind–cull (days)** |
| --- | --- | --- | --- | --- | --- | --- | --- | --- | --- | --- |
| A* | 147825 | F | + | het | wt | wt | 27.00 | 10.86 | 174 | 98 |
| A | 147313 | F | + | het | wt | wt | 15.00 | 9.57 | 277 | 210 |
| A | 147308 | M | + | het | wt | wt | 17.00 | 13.00 | 326 | 235 |
| A | 152008 | M | + | het | wt | wt | 6.00 | 10.43 | 340 | 267 |
| A | 152009 | F | + | het | wt | wt | 23.00 | 12.86 | 394 | 304 |
| A | 152012 | F | + | het | wt | wt | 6.00 | 10.43 | 333 | 260 |
| A | 154127 | F | + | het | wt | wt | 6.00 | 9.14 | 347 | 283 |
| A | 155301 | F | + | het | wt | wt | 6.00 | 8.14 | 363 | 306 |
| A | 156598 | M | + | het | wt | wt | 8.00 | 9.86 | 368 | 299 |
| A | 156599 | M | + | het | wt | wt | 8.00 | 9.86 | 319 | 250 |
| A | 147316 | F | + | het | wt | wt | 18.00 | 9.57 | 338 | 271 |
| A | 147328 | M | + | het | wt | wt | 7.00 | 8.00 | 443 | 387 |
| A | 149821 | M | + | het | wt | wt | 7.00 | 6.43 | 224 | 179 |
| A | 147828 | F | + | het | wt | wt | 30.00 | 11.00 | 385 | 308 |
| AN | 114501 | F | + | het | wt | hom | 7.00 | 12.29 | 203 | 117 |
| AN | 130071 | F | + | het | wt | hom | 22.00 | 11.71 | 237 | 155 |
| AN | 130072 | F | + | het | wt | hom | 11.00 | 10.14 | 169 | 98 |
| AN | 130073 | F | + | het | wt | hom | 22.00 | 11.71 | 208 | 126 |
| AN | 134811 | M | + | het | wt | hom | 5.00 | 9.86 | 215 | 146 |
| AN | 134816 | F | + | het | wt | hom | 60.00 | 18.14 | 298 | 171 |
| AN | 136181 | M | + | het | wt | hom | 5.00 | 9.43 | 263 | 197 |
| AN | 136182 | M | + | het | wt | hom | 5.00 | 9.43 | 243 | 177 |
| AN | 147322 | F | + | het | wt | hom | 27.00 | 10.86 | 203 | 127 |
| AN | 147310 | M | + | het | wt | hom | 7.00 | 8.00 | 184 | 128 |
| AN | 147315 | F | + | het | wt | hom | 30.00 | 11.29 | 223 | 144 |
| AN | 150446 | F | + | het | wt | hom | 17.00 | 11.00 | 375 | 298 |
| AN | 147826 | M | + | het | wt | hom | 17.00 | 12.71 | 231 | 142 |
| AN | 147830 | F | + | het | wt | hom | 17.00 | 12.71 | 232 | 143 |
| AN | 152006 | M | + | het | wt | hom | 6.00 | 10.43 | 214 | 141 |
| AN | 155299 | M | + | het | wt | hom | 6.00 | 8.14 | 200 | 143 |
| AN | 128556* | M | + | het | wt | hom | 9.00 | 8.43 | 287 | 228 |
| AK | 156594 | M | + | het | het | wt | 8.00 | 9.71 | 134 | 66 |
| AK | 156595 | F | + | het | het | wt | 8.00 | 9.71 | 150 | 82 |
| AK | 157868 | F | + | het | het | wt | 21.00 | 11.00 | 171 | 94 |
| AK | 157870 | F | + | het | het | wt | 21.00 | 11.00 | 183 | 106 |
| AK | 171622 | F | + | het | het | wt | 22.00 | 11.86 | 136 | 53 |
| AK | 191820 | M | + | het | het | wt | 16.00 | 7.71 | 119 | 65 |
| AK | 191821 | M | + | het | het | wt | 16.00 | 7.71 | 115 | 61 |
| AK | 191825 | F | + | het | het | wt | 21.00 | 8.57 | 116 | 56 |
| AK | 191827 | F | + | het | het | wt | 21.00 | 8.57 | 118 | 58 |
| AK | 191828 | F | + | het | het | wt | 25.00 | 9.14 | 130 | 66 |
| AKN | 121337 | F | + | het | het | hom | 9.00 | 14.43 | 191 | 90 |
| AKN | 123349 | M | + | het | het | hom | 9.00 | 12.29 | 136 | 50 |
| AKN | 126370 | F | + | het | het | hom | 28.00 | 13.14 | 153 | 61 |
| AKN | 128123 | M | + | het | het | hom | 9.00 | 9.14 | 106 | 42 |
| AKN | 128558 | F | + | het | het | hom | 42.00 | 13.14 | 103 | 11 |
| AKN | 128559 | F | + | het | het | hom | 42.00 | 13.14 | 165 | 73 |
| AKN | 128560 | F | + | het | het | hom | 31.00 | 11.57 | 172 | 91 |
| AKN | 130066 | M | + | het | het | hom | 16.00 | 10.86 | 127 | 51 |
| AKN | 134813 | F | + | het | het | hom | 14.00 | 11.57 | 141 | 60 |
| AKN | 134815 | F | + | het | het | hom | 30.00 | 13.86 | 118 | 21 |
| AKN | 156596 | F | + | het | het | hom | 21.00 | 11.57 | 130 | 49 |
| AKN | 157865 | M | + | het | het | hom | 8.00 | 9.14 | 126 | 62 |

Details of mice in tumour cohorts including identification number (ID), sex, genotype (A = *Apc*, K = *Kras*‑, N = *N-wasp*), days in non-barrier unit prior to induction (Days in unit), age in weeks at induction [Age at ind (weeks)], age when culled [Birth–cull (days)], and survival from induction to cull [Ind–cull (days)]. Vil = Villin-Cre^ERT2^. Cohort: A = *Apc*^fl/+^; AN = *Apc*^fl/+^*N-wasp*^fl/fl^; AK = *Apc*^fl/+^*Kras*^G12D/+^; AKN = *Apc*^fl/+^*Kras*^G12D/+^*N-wasp*^fl/fl^.

*Censored data.

Table S4. Clinicopathological characteristics of the screen-detected cancer cohort

|  |  | **Number (%) *n* = 182** |
| --- | --- | --- |
| Age, years | < 65 | 71 (39.0) |
|  | 65–75 | 89 (48.9) |
|  | > 75 | 22 (12.1) |
| Sex | Female | 55 (30.2) |
|  | Male | 127 (69.8) |
| Site of tumour | Right colon | 26 (14.3) |
|  | Left colon | 108 (59.3) |
|  | Rectum | 48 (26.4) |
| T stage | 1 | 136 (74.7) |
|  | 2 | 46 (25.3) |
|  | 3 | 0 (0) |
|  | 4 | 0 (0) |
| N stage | 0 | 108 (59.3) |
|  | 1 | 14 (7.7) |
|  | 2 | 4 (2.2) |
|  | No data | 56 (30.8) |
| Differentiation | Poor | 5 (2.7) |
|  | Moderate | 169 (92.9) |
|  | Well | 7 (4.3) |
| Vascular invasion | Yes | 46 (25.3) |
|  | No | 129 (70.9) |
|  | No data | 7 (3.8) |

Table S5. Correlation of N-WASP histoscore and clinicopathological characteristics in the screen-detected cancer TMA. *P* values derived from chi-squared test, *n* = 159

| **Parameters** |  | **N-WASP expression score (epithelium)** | | | ***P* value** |
| --- | --- | --- | --- | --- | --- |
|  |  | **Total** | **Low**  **(0–163)** | **High**  **(163.01–300)** |  |
| Gender | Male | 110 | 54 | 56 |  |
|  | Female | 49 | 25 | 24 | 0.822 |
| Age, years | < 65 | 63 | 23 | 40 |  |
|  | 65+ | 96 | 56 | 40 | **0.007** |
| TNM stage | 1 | 142 | 72 | 70 |  |
|  | 2 | 0 | 0 | 0 |  |
|  | 3 | 17 | 7 | 10 | 0.458 |
| Tumour site | Rectum | 43 | 21 | 22 |  |
|  | Left colon | 95 | 47 | 48 |  |
|  | Right colon | 21 | 11 | 10 | 0.963 |
| T stage | 1 | 114 | 62 | 52 |  |
|  | 2 | 45 | 17 | 28 | 0.059 |
| N stage | 0 | 98 | 44 | 54 |  |
|  | 1 | 13 | 6 | 7 |  |
|  | 2 | 4 | 1 | 3 | 0.728 |
| Differentiation | Well/moderate | 155 | 79 | 76 |  |
|  | Poor | 4 | 0 | 4 | **0.044** |
| Vascular invasion | Absent | 109 | 56 | 53 |  |
|  | Present | 45 | 19 | 26 | 0.301 |

Table S6. Clinicopathological characteristics of the non-screen-detected cancer cohort

|  |  | **Number (%) *n* = 272** |
| --- | --- | --- |
| Age, years | < 65 | 97 (36)) |
|  | 65–75 | 89 (33) |
|  | > 75 | 86 (31) |
| Sex | Female | 125 (46) |
|  | Male | 147 (54) |
| Site of tumour | Right colon | 105 (38.6) |
|  | Left colon | 77 (28.3) |
|  | Rectum | 90 (33.1) |
| T stage | 1 | 9 (3.3) |
|  | 2 | 20 (7.4) |
|  | 3 | 162 (59.6) |
|  | 4 | 81 (29.8) |
| N stage | 0 | 152 (55.9) |
|  | 1 | 91 (33.5) |
|  | 2 | 29 (10.7) |
| M stage | 0 | 272 (100) |
|  | 1 | 0 (0) |
| Dukes stage | A | 20 (7.4) |
|  | B | 132 (48.5) |
|  | C | 120 (44.1) |
| No of lymph nodes sampled | < 12 | 93 (34) |
|  | ≥ 12 | 179 (66) |
| Neoadjuvant therapy | Yes | 5 (1.8) |
|  | No | 260 (95.6) |
|  | Missing data | 7 (2.6) |
| Adjuvant therapy | Yes | 81 (29.8) |
|  | No | 191 (70.2) |
| Differentiation | Well/moderate | 239 (87.9) |
|  | Poor | 33 (12.1) |
| Vascular invasion | Yes | 99 (36.4) |
|  | No | 173 (63.6) |
| Margin involvement | Yes | 21 (7.7) |
|  | No | 251 (92.3) |
| Peritoneal involvement | Yes | 80 (29.4) |
|  | No | 192 (70.6) |
| Tumour perforation | Yes | 10 (3.7) |
|  | No | 262 (96.3) |
| Petersen index | High risk | 52 (19.1) |
|  | Low risk | 220 (80.9) |
| Klintrup category | Strong | 90 (33.1) |
|  | Weak | 181 (66.5) |
|  | Missing data | 1 (0.4) |

Table S7. Correlation of N-WASP histoscore and clinicopathological characteristics in the non-screen-detected cancer TMA. *P* values derived from chi-squared test, *n* = 153

| **Parameters** |  | **N-WASP expression score (epithelium)** | | | ***P* value** |
| --- | --- | --- | --- | --- | --- |
|  |  | **Total** | **Low**  **(0–185)** | **High**  **(185–300)** |  |
| Gender | Male | 85 | 47 | 38 |  |
|  | Female | 68 | 31 | 37 | 0.233 |
| Age, years | < 65 | 65 | 47 | 18 |  |
|  | 65–74 | 40 | 30 | 10 |  |
|  | ≥ 75 | 48 | 27 | 21 | 0.555 |
| Site | Rectum | 52 | 24 | 28 |  |
|  | Left colon | 37 | 21 | 16 |  |
|  | Right colon | 64 | 33 | 31 | 0.610 |
| Dukes stage | A | 14 | 8 | 6 |  |
|  | B | 70 | 34 | 36 |  |
|  | C | 69 | 36 | 33 | 0.813 |
| TNM | 1 | 14 | 8 | 6 |  |
|  | 2 | 69 | 33 | 36 |  |
|  | 3 | 70 | 37 | 33 | 0.746 |
| T stage | 1 | 4 | 4 | 0 |  |
|  | 2 | 15 | 7 | 8 |  |
|  | 3 | 90 | 42 | 48 |  |
|  | 4 | 44 | 25 | 19 | 0.156 |
| N stage | 0 | 84 | 42 | 42 |  |
|  | 1 | 54 | 30 | 24 |  |
|  | 2 | 15 | 6 | 9 | 0.547 |
| Differentiation | Well/moderate | 136 | 68 | 68 |  |
|  | Poor | 17 | 10 | 7 | 0.493 |
| Venous invasion | Absent | 92 | 46 | 46 |  |
|  | Present | 61 | 32 | 29 | 0.766 |
| Peritoneal involvement | No | 108 | 54 | 54 |  |
|  | Yes | 45 | 24 | 21 | 0.707 |
| Tumour perforation | No | 147 | 73 | 74 |  |
|  | Yes | 6 | 5 | 1 | 0.106 |
| Petersen index | Low (0–1) | 121 | 58 | 63 |  |
|  | High (2–5) | 32 | 20 | 12 | 0.143 |
| Klintrup category | Weak | 100 | 51 | 49 |  |
|  | Strong | 52 | 27 | 25 | 0.914 |
| MMR deficiency | No | 131 | 61 | 70 |  |
|  | Yes | 22 | 17 | 5 | **0.008** |
